# Supplementary material for: Alternative splicing coupled to nonsense-mediated decay coordinates downregulation of non-neuronal genes in developing mouse neurons
Source: Genome Biol. 2024 Jun 20;25:162. doi: 10.1186/s13059-024-03305-8 (PMC11188260; doi:10.1186/s13059-024-03305-8)
Supplement: Supplementary file 1 — Additional file 1. Supplementary Figures. [file 13059_2024_3305_MOESM1_ESM.pdf]

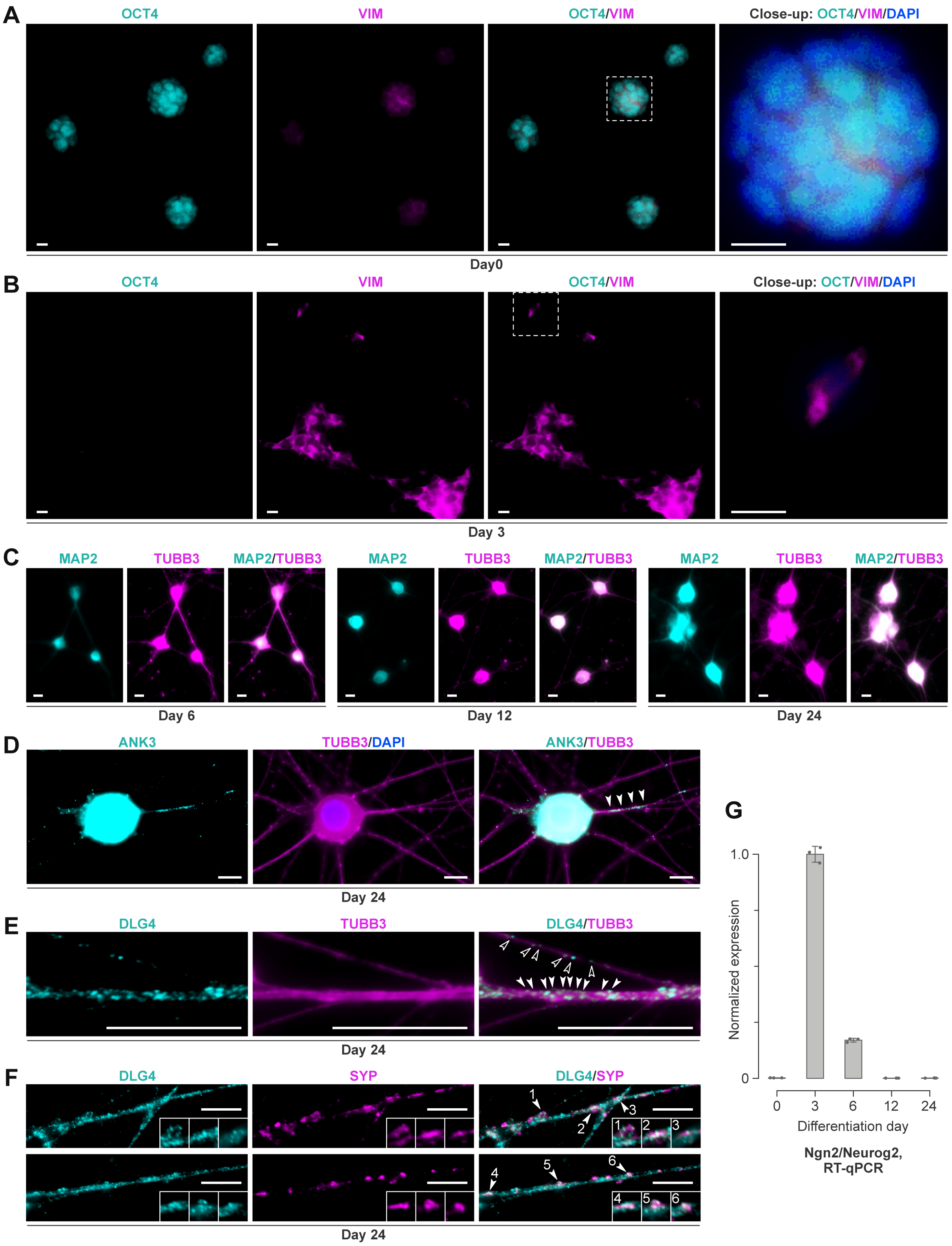

Fig. S1: Expression of developmental markers in Dox-induced TRE-Ngn2 cultures.

(A-B) TRE-Ngn2 cells fixed on differentiation day 0 (A) or day 3 (B) were immunostained for the ESC marker OCT4/POU5F1 (Abcam, ab19857; 1:500) and the NPC-enriched marker VIM (Abcam, ab8978; 1:500). Note that OCT4 is expressed in day-0 but not day-3 cells and that VIM is strongly upregulated on day 3. Dashed rectangles show areas magnified in the close-ups

(C) TRE-Ngn2 samples differentiated for 6-24 days were immunostained for an early neuronal marker, TUBB3/tubulin βIII (Covance, PRB-435P; 1:5000), and a later neuronal marker, MAP2 (Covance, PCK-554P, 1:1000). Note an increase in MAP2 expression compared to the TUBB3 signal at later stages of differentiation.

(D-F) Day-24 TRE-Ngn2 cultures were stained for mature neuronal markers: (D) ANK3/ANKG (NeuroMab, 75-146, 1:500) and (E-F) DLG4/PSD-95 (NeuroMab, 75-028, 1:500) and SYP/Synaptophysin (Thermo Fisher Scientific, 18-0130, 1:500). Samples in (D-E) were additionally co-stained for TUBB3 to visualize overall neuronal morphology. Arrowheads in (D) indicate an ANK3-positive axon initial segment, and in (E), DLG4 puncta in individual neurites (open arrowheads) and neurite bundles (solid arrowheads). Panel (F) shows two examples of neuropil regions, with arrowheads indicating SYP-positive presynaptic sites juxtaposed in neurite bundles to DLG4-positive postsynaptic puncta. These structures are magnified 2-fold in the close-ups. (A-F) Scale bars, 10 μm.

(**G**) RT-qPCR analysis shows that the *Ngn2*/*Neurog2* expression in differentiating TRE-Ngn2 cells peaks at the NPC stage (day 3) and declines at later stages. This mimics the natural regulation of *Ngn2* in neurodevelopment [45].

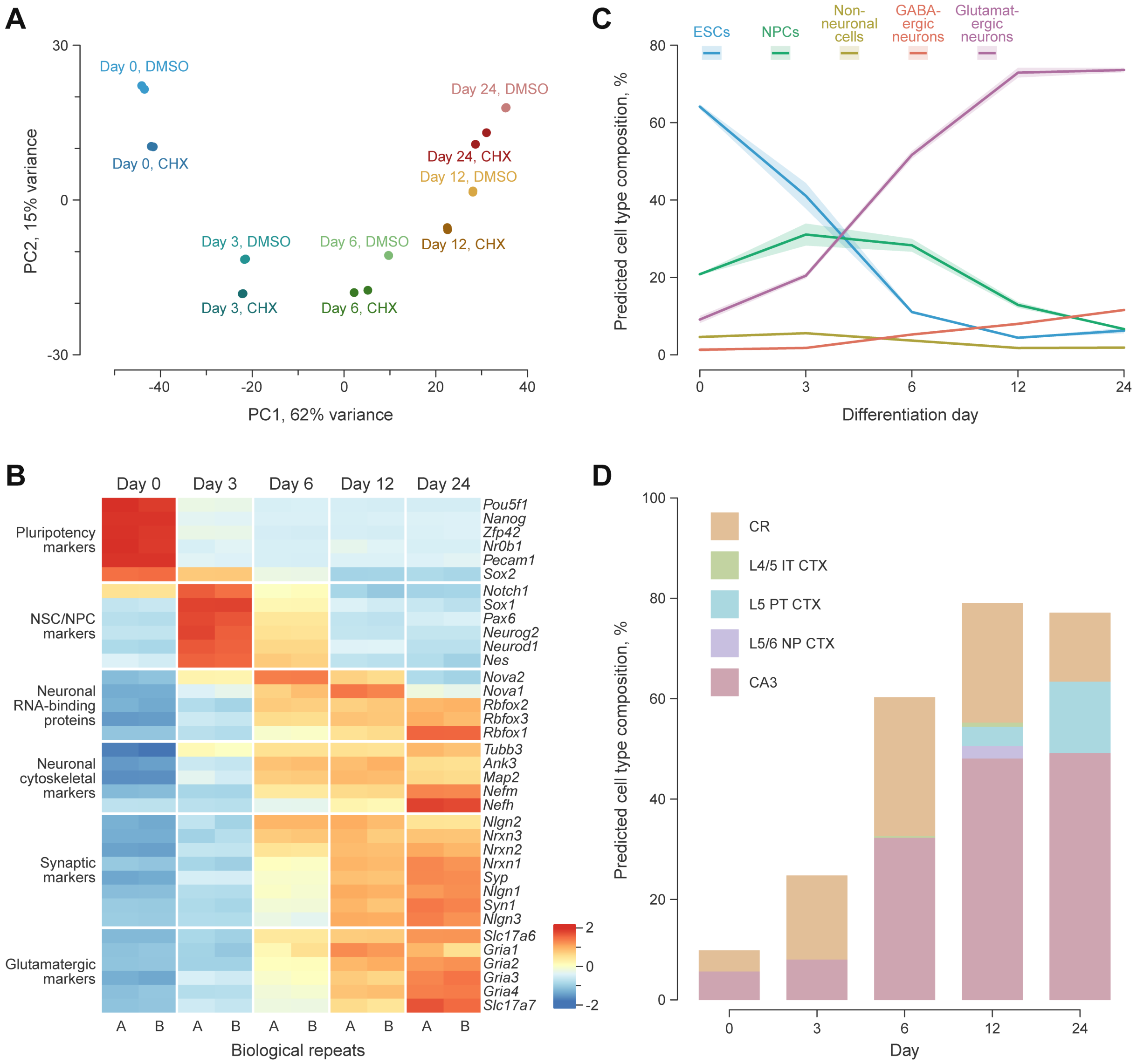

Fig. S2: Initial characterization of the TRE-Ngn2 RNA-seq data.

(A) Principal component analysis (PCA) showing that the transcriptome of TRE-Ngn2 cells changes as a function of development and responds to the CHX treatment.

(B) Heat map analysis of stage-specific markers in the control-treated TRE-Ngn2 RNA-seq samples.

(C) Changes in the cellular composition of differentiating TRE-Ngn2 cultures deconvolved by MuSiC [48].

(D) Glutamatergic neuron subtype-specific gene expression signatures detected by MuSiC. CR, Cajal-Retzius neurons; L4/5 IT CTX, cortical layer 4/5 intratelencephalic neurons; L5 PT CTX, cortical layer 5 pyramidal tract neurons; L5/6 NP CTX, cortical layer 5/6 near-projecting neurons; CA3, hippocampal CA3 neurons.

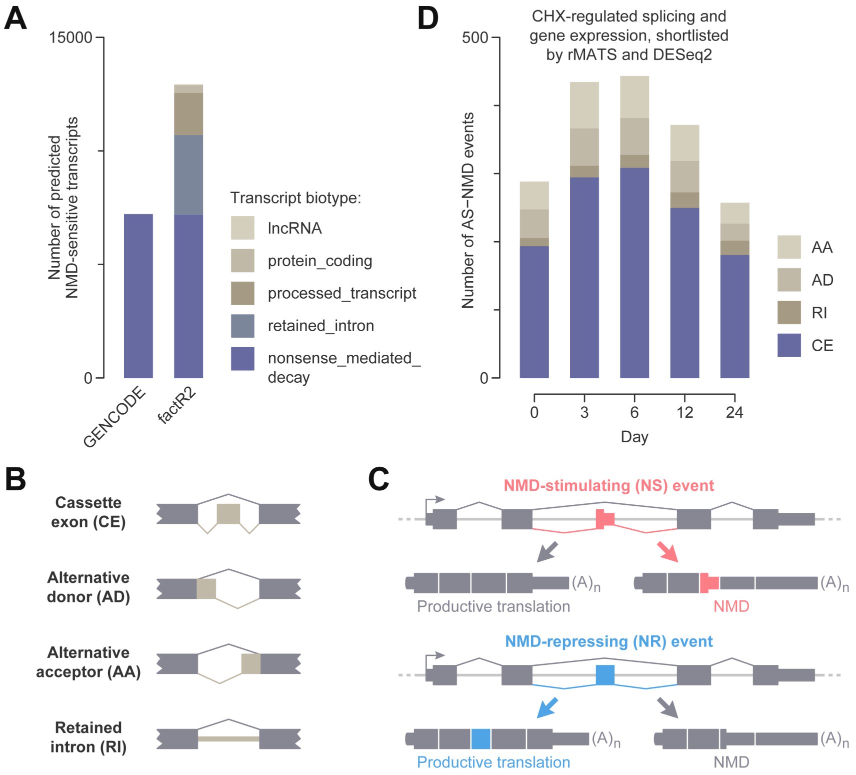

Fig. S3: factR2 analysis of AS-NMD candidates.

(**A**) Transcript biotypes for AS-NMD candidates identified by reanalyzing the GENCODE transcriptome using factR2.

(B) The key types of alternatively spliced events examined by factR2.

(C) The opposite effects of NMD-stimulating (NS) and NMD-repressing (NR) events on gene expression illustrated for cassette exons, as an example.

(D) Distribution of AS-NMD events responding to CHX at both the splicing and gene expression levels at different stages of neuronal differentiation. The custom transcriptome was analyzed as described in Fig. 1E, except using rMATS with $|\Delta PSI|>0.1$ and $FDR<0.05$ cutoffs to shortlist regulated splicing events.

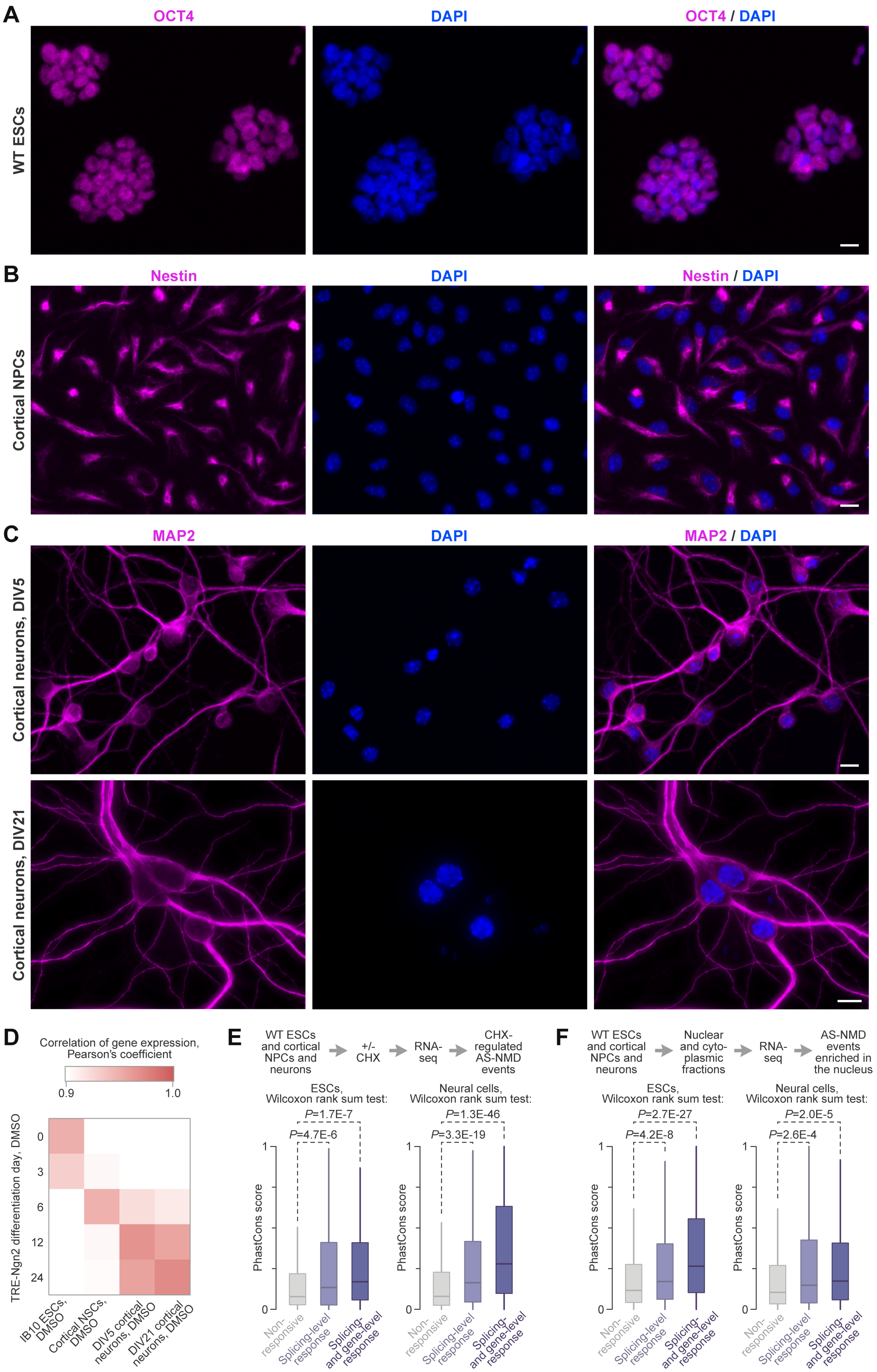
A
OCT4
DAPI
OCT4 / DAPI
WT ESCs
B
Nestin
DAPI
Nestin / DAPI
Cortical NPCs
C
MAP2
DAPI
MAP2 / DAPI
Cortical neurons, DIV5
Cortical neurons, DIV21
D
Correlation of gene expression, Pearson's coefficient
0.9
1.0
TRE-Ngn2 differentiation day, DMSO
0
3
6
12
24
IB10 ESCs, DMSO
Cortical NSCs, DMSO
DIV5 cortical neurons, DMSO
DIV21 cortical neurons, DMSO
E
WT ESCs and cortical NPCs and neurons
+/- CHX
RNA-seq
CHX-regulated AS-NMD events
ESCs, Wilcoxon rank sum test:
P=1.7E-7
P=4.7E-6
Neural cells, Wilcoxon rank sum test:
P=1.3E-46
P=3.3E-19
PhastCons score
Non-responsive
Splicing-level response
Splicing- and gene-level response
F
WT ESCs and cortical NPCs and neurons
Nuclear and cyto-plasmic fractions
RNA-seq
AS-NMD events enriched in the nucleus
ESCs, Wilcoxon rank sum test:
P=2.7E-27
P=4.2E-8
Neural cells, Wilcoxon rank sum test:
P=2.0E-5
P=2.6E-4
PhastCons score
Non-responsive
Splicing-level response
Splicing- and gene-level response

Fig. S4: Detailed analyses of wild-type ESCs and primary neural cells.

(A-C) Immunofluorescence analyses of (A) wild-type ESCs (WT; IB10 line) for OCT4 expression; (B) cortical NPCs for NES/Nestin expression; and (C) cortical neurons cultured for 5 or 21 days in vitro (DIV5 and DIV21) for MAP2 expression. Scale bars, 10 μm.

(**D**) Pairwise correlation of total gene expression between natural samples and differentiating TRE-Ngn2 cells.

(E) Interspecies conservation of intronic sequence context of the AS-NMD events responding to CHX in the WT ESCs or primary neural cells and additionally present in different groups of the TRE-Ngn2 events. Higher PhastCons scores [96] indicate stronger conservation across vertebrates. See Fig. 1F for further details.

(F) Interspecies conservation of intronic sequence context of the AS-NMD events enriched in the nucleus of the WT ESCs or primary neural cells and additionally present in different groups of the TRE-Ngn2 events. See Fig. 1G for further details.

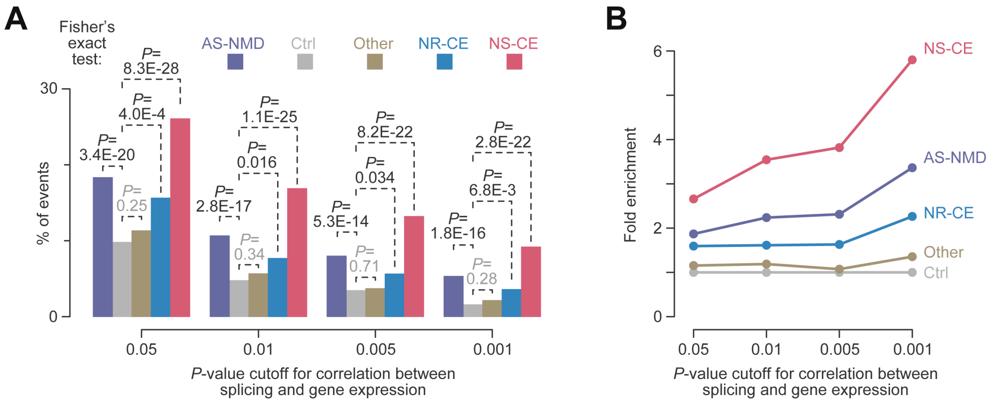

Fig. S5: factR2 events responding to CHX at both the splicing and gene expression levels correlate with developmental changes in gene expression.

The two panels show analyses similar to those presented in Fig. 2A-B, respectively, but using more stringent Whippet filters to define CHX-responsive factR2 events: $|\Delta PSI|>0.25$ instead of >0.1 and probability>0.95 instead of >0.9.

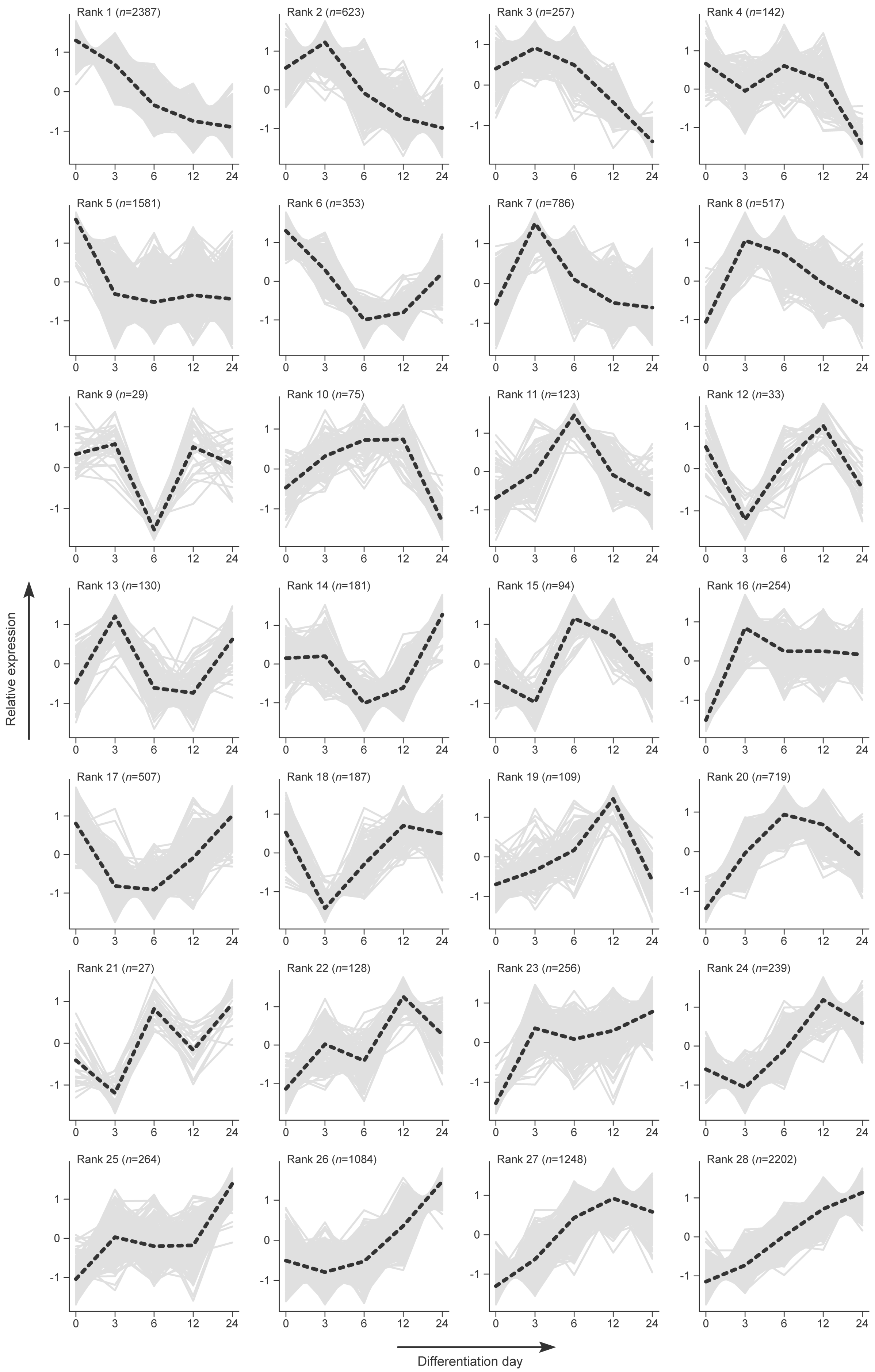

Rank 1 (n=2387)
Rank 2 (n=623)
Rank 3 (n=257)
Rank 4 (n=142)
Rank 5 (n=1581)
Rank 6 (n=353)
Rank 7 (n=786)
Rank 8 (n=517)
Rank 9 (n=29)
Rank 10 (n=75)
Rank 11 (n=123)
Rank 12 (n=33)
Rank 13 (n=130)
Rank 14 (n=181)
Rank 15 (n=94)
Rank 16 (n=254)
Rank 17 (n=507)
Rank 18 (n=187)
Rank 19 (n=109)
Rank 20 (n=719)
Rank 21 (n=27)
Rank 22 (n=128)
Rank 23 (n=256)
Rank 24 (n=239)
Rank 25 (n=264)
Rank 26 (n=1084)
Rank 27 (n=1248)
Rank 28 (n=2202)
Relative expression
Differentiation day

Fig. S6: Ranking of gene expression trajectories in differentiating TRE-Ngn2 cells.
Longitudinal RNA-seq data for control-treated TRE-Ngn2 cells were analyzed by DP_GP_cluster [55] and ranked from monotonic downregulation (rank 1) to monotonic upregulation (rank 28) based on the cluster-specific Kendall's trend τ values (see also Fig. S7A). Solid gray lines, temporal dynamics of individual genes in each cluster. Dashed black lines, gene cluster trajectories.

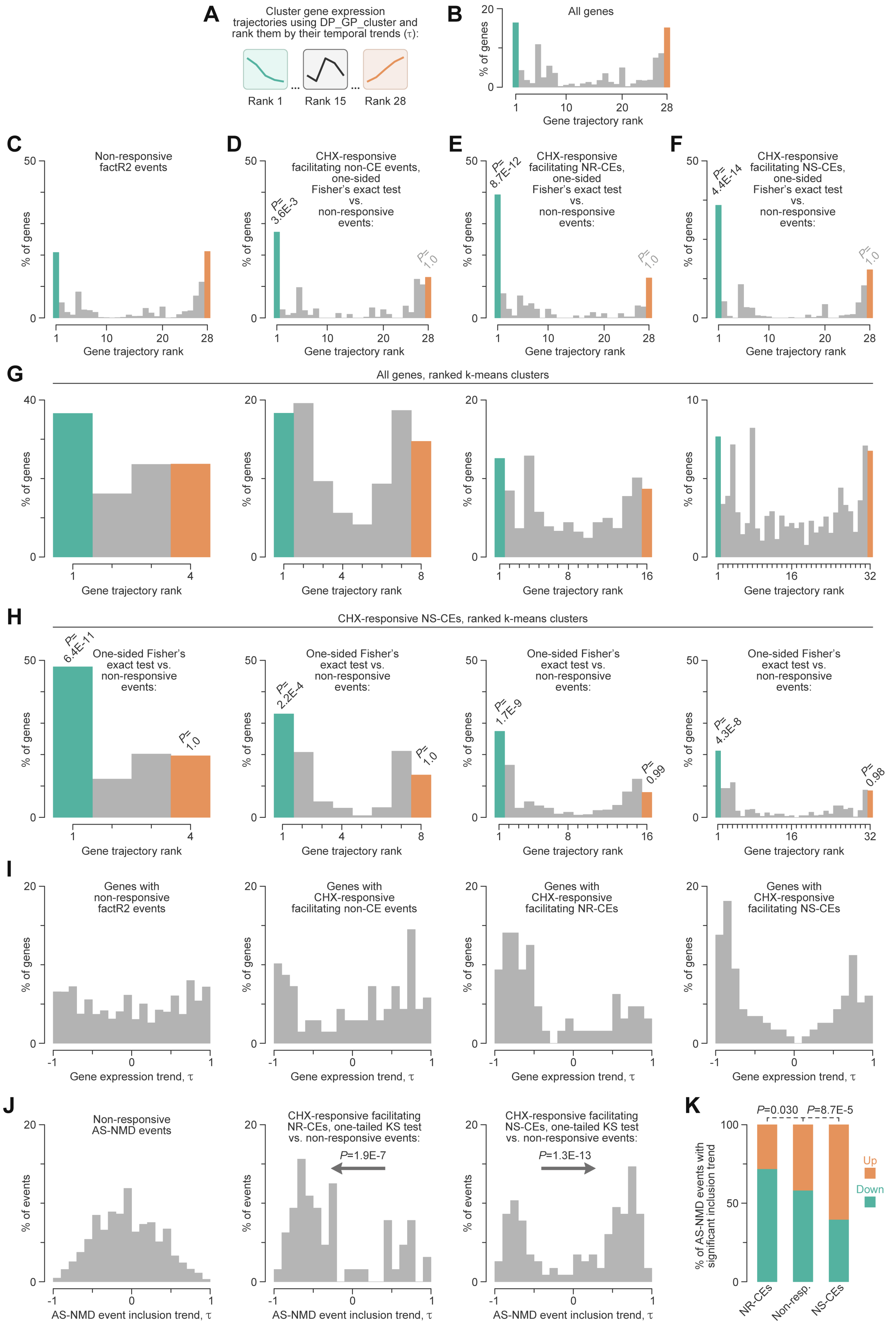

Fig. S7: AS-NMD control of neurodevelopmentally downregulated genes.

(A) Outline of the DP_GP_cluster-based [54] gene expression trajectory analysis workflow.

(B) Distribution of ranked DP_GP_cluster trajectories for all regulated genes (DESeq2's LRT test, FDR<0.001).

(C-F) Ranked DP_GP_cluster trajectories for regulated genes containing (C) only non-responsive factR2 events; (D) CHX-responsive facilitating AD, AA and RI events; (E) CHX-responsive facilitating NR-CEs; (F) CHX-responsive facilitating NS-CEs. The rank-1 (monotonic downregulation) and rank-28 (monotonic upregulation) trajectories were compared to the non-responsive AS-NMD control by one-sided Fisher's exact test. Note that monotonic downregulation is particularly prevalent in (E-F).

(G-H) Gene expression trajectories were clustered using the k-means method and ranked based on their Kendall's trend τ values. The distributions of ranked clusters for k values 4, 8, 16, and 32 were then plotted for (G) all genes and (H) genes containing CHX-responsive facilitating NS-CEs. Note that monotonically downregulated clusters are significantly over-represented in (H) regardless of the k value. Enrichments were calculated using one-sided Fisher's exact test, as described in (F).

(I) Distribution of Kendall's trend τ for individual genes with the indicated types of factR2 events. The prominent peaks of negative τ values in the [-1, -0.75] interval for genes with facilitating NR-CEs and NS-CEs suggest that these groups are often monotonically downregulated in developing neurons.

(J) Neurodevelopmental changes in AS-NMD event inclusion status calculated as Kendall's τ for PSI trend. Note significant overrepresentation of the decreasing trend among CHX-responsive facilitating NR-CEs (middle) and the increasing trend among CHX-responsive facilitating NS-CEs (right), compared to non-responsive AS-NMD events (left). The *P*-values were calculated by one-sided Kolmogorov-Smirnov (KS) test.

(K) Preferential decrease in NR-CE inclusion and increase in NS-CE inclusion in developing neurons is also detectable by Fisher's exact test analysis of events with statistically significant Kendall's trends (*P*<0.05).

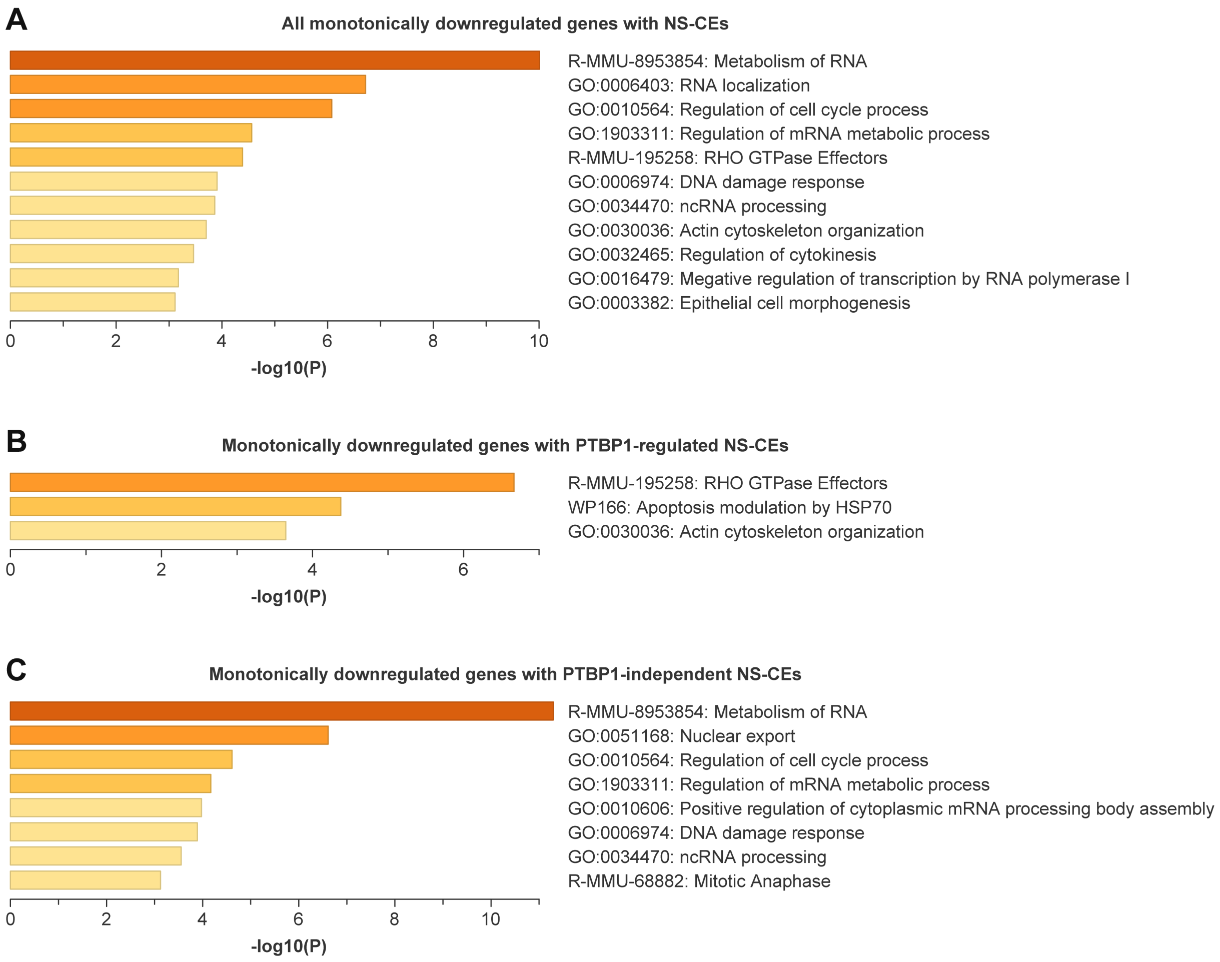

Fig. S8: Neurodevelopmentally downregulated genes with facilitating NS-CEs are enriched for specific biological functions.

Shown are significantly enriched (P<0.001) functional group summaries identified by Metascape [56] for **(A)** all monotonically downregulated genes (Kendall's τ>0.75, P<0.05) containing NS-CEs; **(B)** monotonically downregulated genes with PTBP1-regulated NS-CEs; and **(C)** monotonically downregulated genes with PTBP1-independent NS-CEs. See Additional file 6: Table S5 for further details.

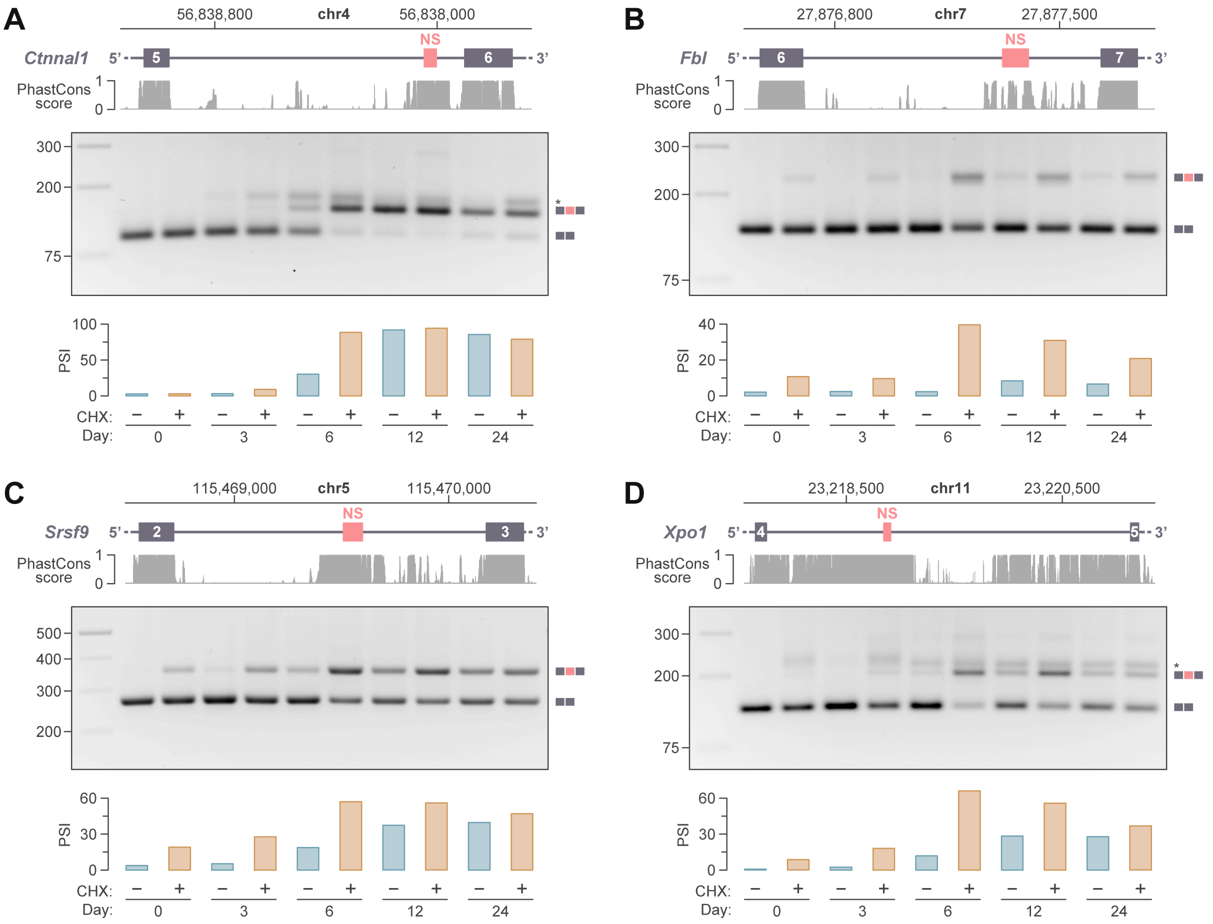

Fig. S9: Experimental validation of facilitating NS-CEs encoded in genes monotonically downregulated in developing neurons.

TRE-Ngn2 cultures were briefly treated with DMSO (odd samples) or CHX (even samples) on differentiation days 0-24 and analyzed by RT-PCR with primers flanking NS-CEs in (A) *Ctnnal1*, (B) *Fbl*, (C) *Srsf9*, and (D) *Xpo1* genes. In each panel, the NS-CE containing parts of genes and PhastCons vertebrate conservation tracks are shown on the top; gel analyses of the RT-PCR products are in the middle; and quantifications of NS-CE percent spliced in (PSI) values in DMSO (blue) and CHX (orange) treated samples are at the bottom. Note that in all four cases, NS-CE inclusion tends to increase as a function of development.

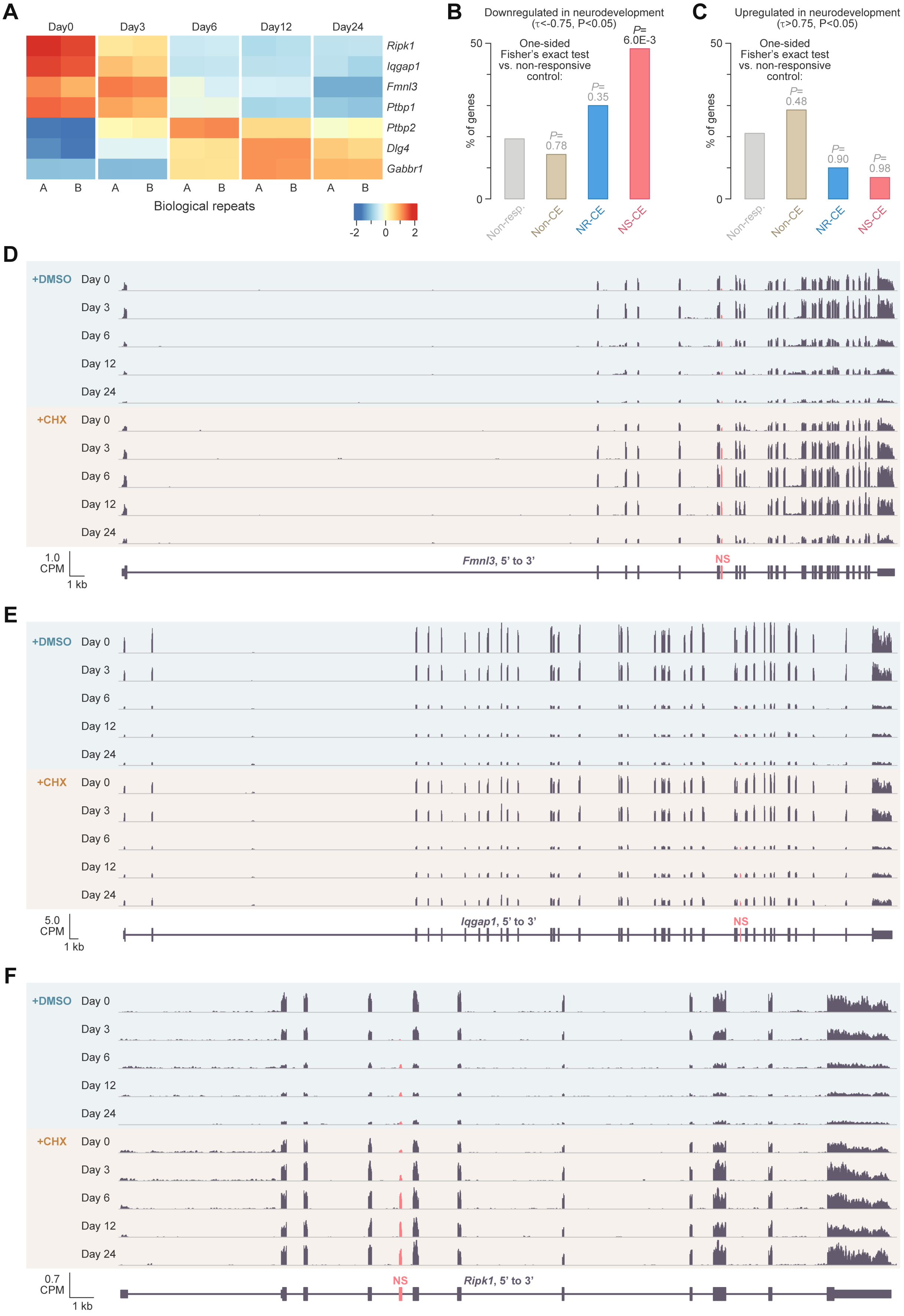

Fig. S10: Examples of PTBP1-regulated AS-NMD target genes.

(A) The expression dynamics of *Ptbp1* and PTBP1-cotrolled targets in differentiating TRE-Ngn2 cells. The targets shown include neurodevelopmentally upregulated genes with previously characterized NR-CEs (*Ptbp2*, *Dlg4*, and *Gabbr1*) and neurodevelopmentally downregulated genes with NS-CEs shortlisted in this study (*Fmnl3*, *Iqgap1*, and *Ripk1*) (Additional file 5: Table S4).

(B) One-tailed Fisher's exact test showing that the monotonic downregulation trend (Kendall's $\tau<-0.75$, $P<0.05$) is overrepresented among genes with PTBP1-controlled facilitating NS-CEs compared to genes containing only non-responsive factR2 events.

(C) Conversely, monotonic upregulation (Kendall's $\tau>0.75$, $P<0.05$) is not enriched among genes with different types of PTBP1-controlled AS-NMD events.

(D-F) Count-per-million (CPM) normalized RNA-seq coverage plots for (D) *Fnml3*, (E) *Iqgap1*, and (F) *Ripk1*. The NS-CEs identified by factR2 are highlighted in red. All three genes are strongly downregulated in control-treated differentiating TRE-Ngn2 samples, and this effect is partially rescued by CHX.

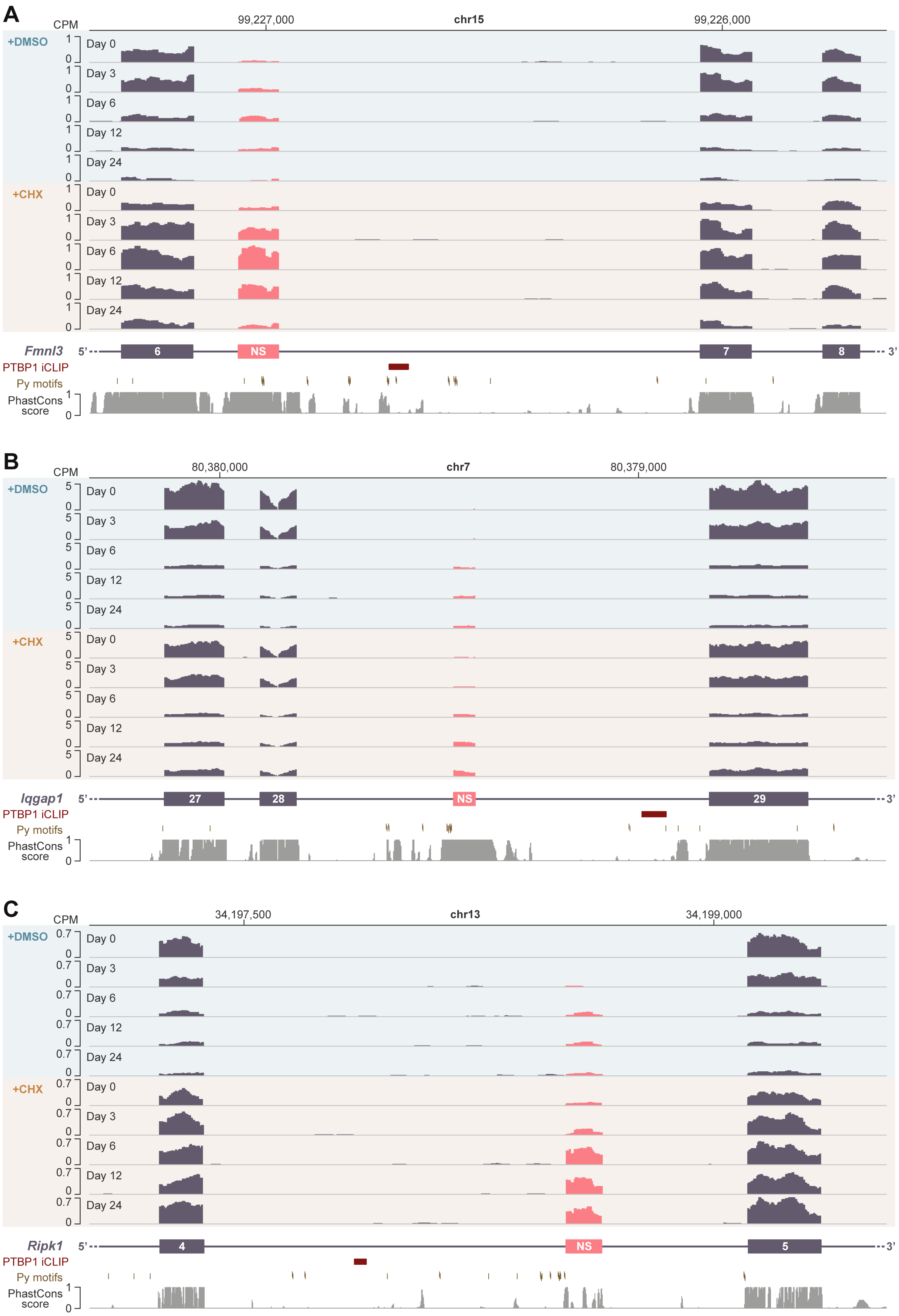

Fig. S11: Alternatively spliced parts of neurodevelopmentally downregulated PTBP1 targets.

Shown are CPM-normalized RNA-seq coverage plots for the regulated parts of **(A)** *Fnml3*, **(B)** *Iqgap1* and **(C)** *Ripk1*. The NS-CEs are highlighted in red. PTBP1 iCLIP clusters [57] (maroon), PTBP1-specific motifs (Py; YTCTYY and YYTCTY; olive brown), and vertebrate PhastCons tracks (gray) are shown at the bottom of each panel. The inclusion of NS-CEs increases as a function of development, and this effect is especially prominent in the CXH-treated series.

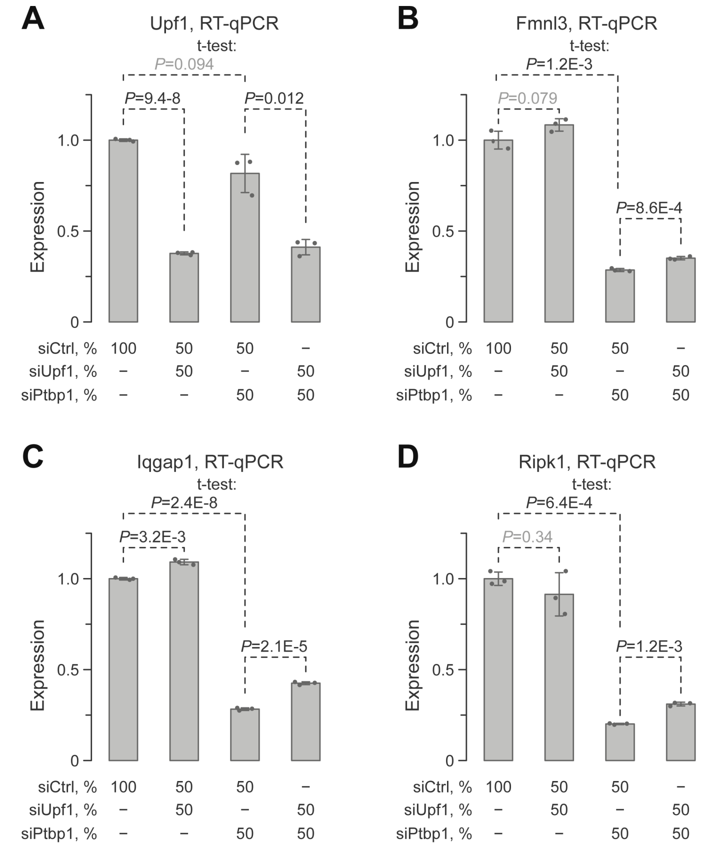

Fig. S12: PTBP1 downregulation dampens the expression of *Fmnl3*, *Iqgap1* and *Ripk1* in an NMD-dependent manner.

Mouse ESCs were treated for 48 hours with siRNAs indicated at the bottom of each panel and analyzed by RT-qPCR with primers against constitutively spliced parts of **(A)** *Upf1*, **(B)** *Fmnl3*, **(C)** *Iqgap1*, and **(D)** *Ripk1*. Note that PTBP1 knockdown dampens the expression of its target genes and that the knockdown of the key NMD factor UPF1 partially rescues this effect.

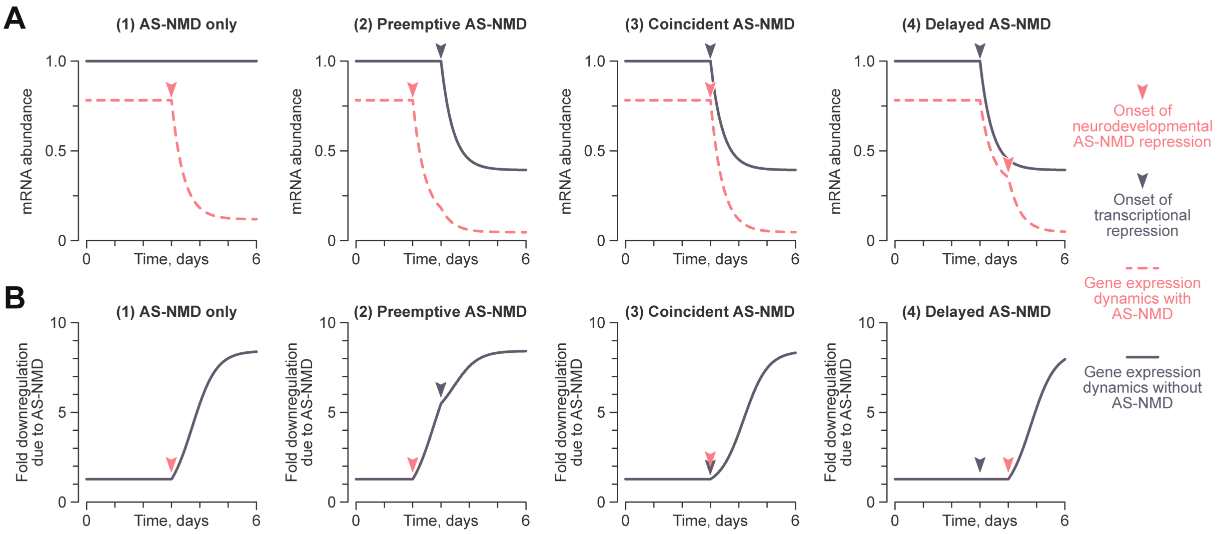

Fig. S13: Possible effects of AS-NMD on gene downregulation in developing neurons.
We used the ordinary differential equation model described in Materials and Methods to estimate AS-NMD contribution to gene expression dynamics when it (1) is the only downregulation mechanism or (2-4) functions alongside transcriptional repression with different onset times. **(A)** Gene expression dynamics with (red) and without (mauve) AS-NMD for the four regulation possibilities. **(B)** Effect of AS-NMD on gene expression calculated from (A) by normalizing the without-AS-NMD trajectories by their with-AS-NMD counterparts.

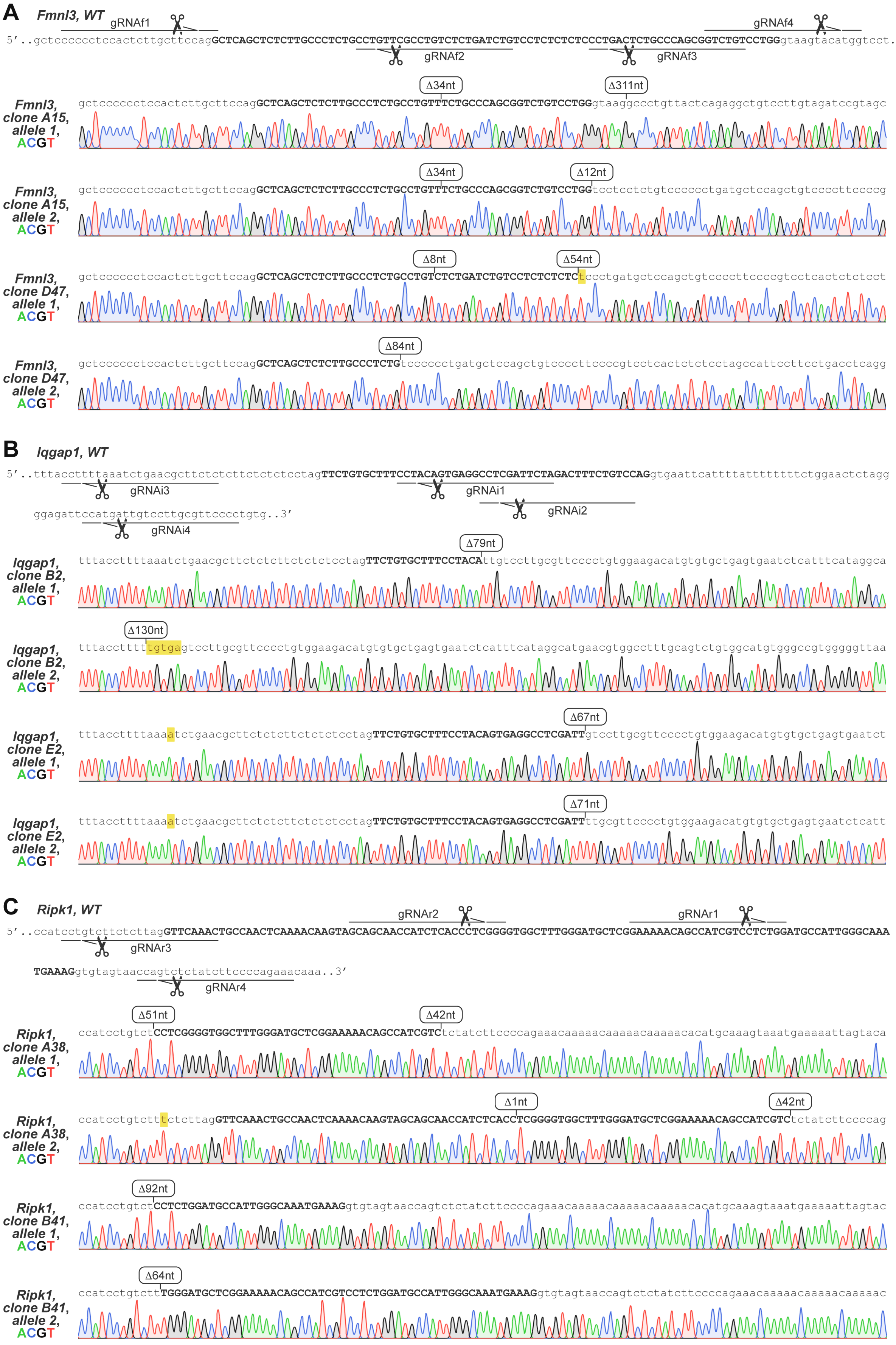

Fig. S14: CRISPR/Cas9 disruption of PTBP1-regulated NS-CEs.

TRE-Ngn2 ESCs were transfected with the expression constructs encoding Cas9 and four different CRISPR gRNAs targeting the NS-CEs and their adjacent intronic sequences in **(A)** *Fmnl3*, **(B)** *Iqgap1*, and **(C)** *Ripk1*. Two clones with biallelic mutations in the NS-CE region were selected for each gene and analyzed by Sanger sequencing. Each panel shows the wild-type gene sequence and the gRNA positions at the top and allele-specific sequencing chromatograms for the two mutant clones at the bottom. Upper case, wild-type NS-CEs and their remnants. Lower case, intronic sequences. Rounded rectangles, CRISPR/Cas9-induced deletions. Yellow highlight, insertions and nucleotide substitutions.

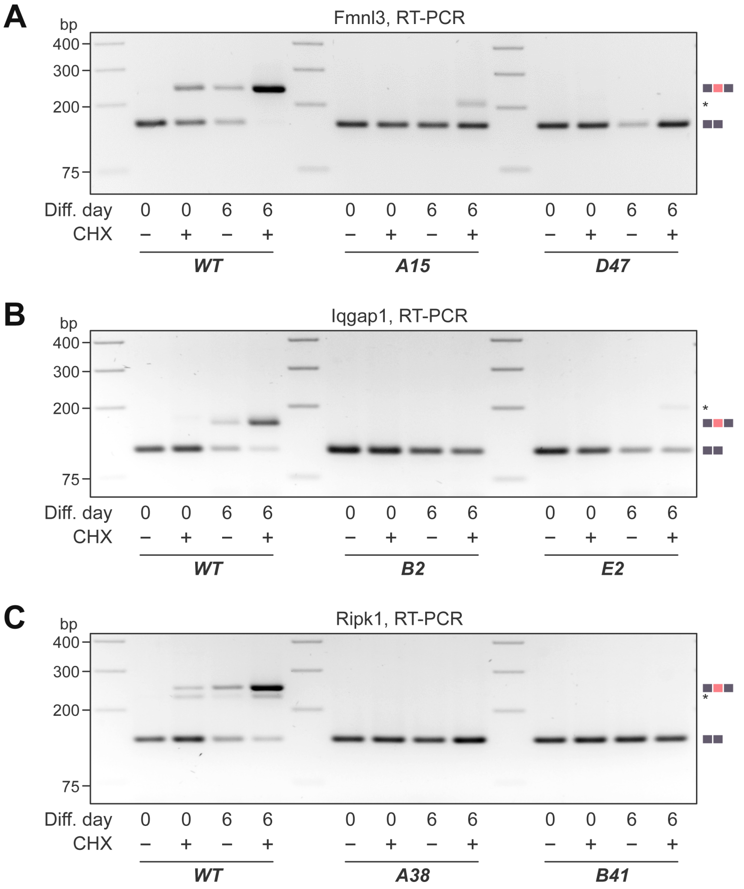

Fig. S15: RT-PCR analysis of wild-type and mutant TRE-Ngn2 cells.
Wild-type (WT) TRE-Ngn2 cells and the NS-CE mutants introduced in Fig. S14 were treated with DMSO or CHX on differentiation days 0 (ESCs) or 6 (young neurons) and analyzed by RT-PCR with **(A)** *Fmnl3*-, **(B)** *Iqgap1*- or **(C)** *Ripk1*-specific primers designed against the NS-CE-flanking exons. In the wild type, NS-CE-containing mRNAs are readily detectable on day 6 in DMSO-treated samples, becoming a major splice form in the presence of CHX. The ability to produce NMD-sensitive mRNA isoforms is either completely lost (clones D47, B2, A38 and B41) or drastically impaired (clones A15 and E2) in the mutant cells. Asterisks, unspecific amplification products and low-abundance NMD-sensitive transcripts detectable in clones A15 and E2.

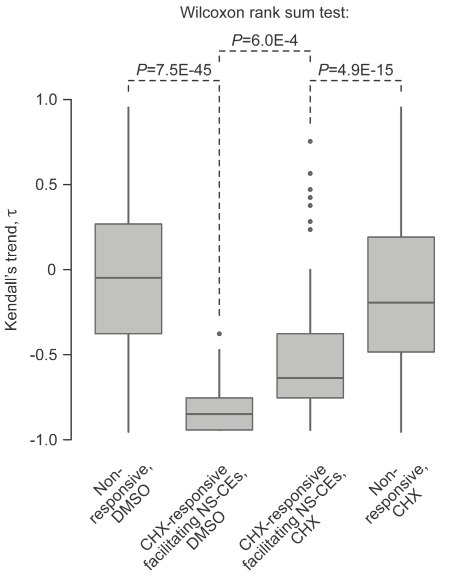

Fig. S16: Neurodevelopmentally downregulated NS-CE-containing genes tend to maintain downward expression trajectories (negative Kendall's trend τ values) in the presence of CHX.

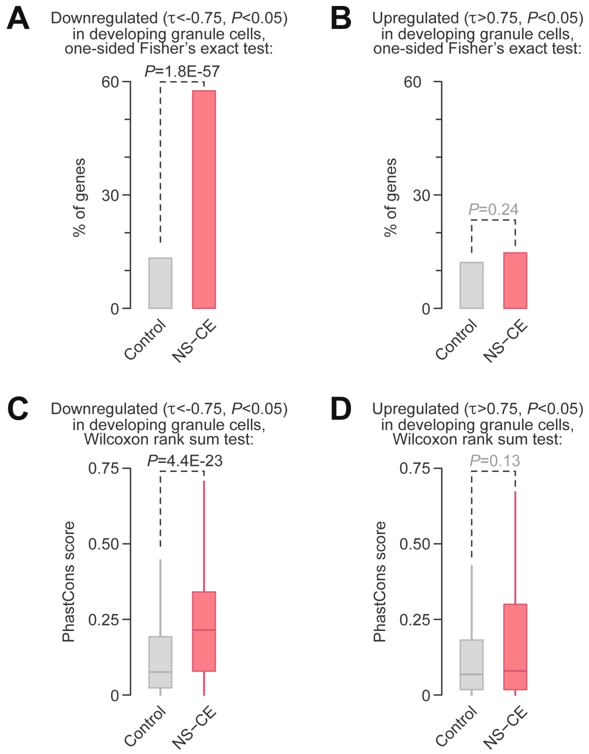

Fig. S17: NS-CEs provide a widespread mechanism potentiating gene downregulation in developing neurons.

(A-B) High-quality NS-CE events identified by factR2 analysis of developing dentate gyrus granule cells [68] are enriched in developmentally downregulated (A) but not upregulated (B) genes compared to CE-containing genes not predicted by factR2 to undergo AS-NMD.

(C-D) The granule-cell NS-CEs encoded in developmentally downregulated, but not upregulated genes tend to be conserved [96] significantly stronger compared to the corresponding groups of CEs not predicted by factR2 to be involved in AS-NMD.
