# Supplementary material for: Alternative splicing coupled to nonsense-mediated decay coordinates downregulation of non-neuronal genes in developing mouse neurons
Source: Genome Biol. 2024 Jun 20;25:162. doi: 10.1186/s13059-024-03305-8 (PMC11188260; doi:10.1186/s13059-024-03305-8)
Supplement: Supplementary file 7 — Additional file 7. Supplementary Results. [file 13059_2024_3305_MOESM7_ESM.docx]

***NS-CEs may contribute to gene downregulation in other developmental contexts***

The NS-CEs investigated in the main text were identified in developing glutamatergic neurons. To examine the incidence of NS-CEs in a different system, we performed factR2 analysis for developing dentate gyrus granule cells (RNA-seq data from [68]), which are known to secrete a combination of neurotransmitters [69]. Since the granule cell dataset lacked the CHX treatment, we used facilitation (i.e. a positive correlation between splicing patterns protecting mRNA from NMD and gene expression) as the sole criterion to shortlist 235 NS-CE candidates (Table S6). The rationale for this approach was that CHX-responsive NS-CEs were enriched for the facilitating behavior in our TRE-Ngn2 analysis (Fig. 2A-B). Consistent with the cell type-specific differences in gene expression, many of the granule cell events were novel compared to their TRE-Ngn2 counterparts. An interesting shared example was the Bak1 NS-CE previously shown to dampen the expression of this pro-apoptotic factor in developing neurons [32].

Strikingly, nearly 60% of the granule cell genes containing shortlisted NS-CEs were monotonically downregulated during granule development (Kendall's τ<-0.75, *P*<0.05). This constituted a ~4.3-fold enrichment compared to a control group containing CEs not associated with AS-NMD (Fig. S17A). We did not detect such enrichment for monotonically upregulated genes (Fig. S17B). Notably, intronic sequence context of NS-CEs in monotonically downregulated but not upregulated genes showed a stronger interspecies conservation compared to non-AS-NMD CEs (Fig. S17C-D).

These analyses indicate that NMD-stimulating cassette exons are widespread and evolutionarily conserved regulatory elements involved in gene downregulation in developing neurons.
