# Supplementary material for: Alternative splicing coupled to nonsense-mediated decay coordinates downregulation of non-neuronal genes in developing mouse neurons
Source: Genome Biol. 2024 Jun 20;25:162. doi: 10.1186/s13059-024-03305-8 (PMC11188260; doi:10.1186/s13059-024-03305-8)
Supplement: Supplementary file 12 — Additional file 12. Table S10. Media for induced neuronal differentiation of TRE-Ngn2 cells. [file 13059_2024_3305_MOESM12_ESM.docx]

**Table S10**: Media for induced neuronal differentiation of TRE-Ngn2 cells

**iN-D0 medium**

| **Reagent** | **Final concentration** | **Manufacturer** | **Cat#** |
| --- | --- | --- | --- |
| Neurobasal | 0.5× | Thermo Fisher Scientific | 21103049 |
| DMEM/F12 | 0.5× | Sigma | D6421 |
| PenStrep | 100 units/ml | Thermo Fisher Scientific | 15140122 |
| N2 | 1× | Gibco | 17502048 |
| B27 with retinoic acid | 1× | Gibco | 17504044 |
| Laminin | 1 µg/ml | Sigma | L2020 |
| Insulin | 20 µg/ml | Sigma | I0516 |
| L-glutamine | 500 µM | Gibco | 25030-024 |
| db-cAMP | 2 mM | Sigma | D0627 |
| NT-3 | 10 ng/ml | Miltenyi Biotec | 130-093-973 |
| β-mercaptoethanol | 100 µM | Sigma | M3148 |
| Doxycycline | 2 μg/ml | Sigma | D9891 |

**iN-D2 medium**

| **Reagent** | **Final concentration** | **Manufacturer** | **Cat#** |
| --- | --- | --- | --- |
| Neurobasal | 0.5× | Thermo Fisher Scientific | 21103049 |
| DMEM/F12 | 0.5× | Sigma | D6421 |
| PenStrep | 100 units/ml | Thermo Fisher Scientific | 15140122 |
| N2 | 1× | Gibco | 17502048 |
| B27 with retinoic acid | 1× | Gibco | 17504044 |
| Laminin | 1 µg/ml | Sigma | L2020 |
| Insulin | 20 µg/ml | Sigma | I0516 |
| L-glutamine | 500 µM | Gibco | 25030-024 |
| db-cAMP | 2 mM | Sigma | D0627 |
| NT-3 | 10 ng/ml | Miltenyi Biotec | 130-093-973 |
| L-ascorbic acid | 200 µM | Sigma | A4403 |
| Doxycycline | 1 μg/ml | Sigma | D9891 |

**iN-D4 medium**

| **Reagent** | **Final concentration** | **Manufacturer** | **Cat#** |
| --- | --- | --- | --- |
| Neurobasal | 0.5× | Thermo Fisher Scientific | 21103049 |
| DMEM/F12 | 0.5× | Sigma | D6421 |
| PenStrep | 100 units/ml | Thermo Fisher Scientific | 15140122 |
| N2 | 1× | Gibco | 17502048 |
| B27 with retinoic acid | 1× | Gibco | 17504044 |
| Laminin | 1 µg/ml | Sigma | L2020 |
| Insulin | 20 µg/ml | Sigma | I0516 |
| L-glutamine | 500 µM | Gibco | 25030-024 |
| db-cAMP | 2 mM | Sigma | D0627 |
| NT-3 | 10 ng/ml | Miltenyi Biotec | 130-093-973 |
| L-ascorbic acid | 200 µM | Sigma | A4403 |

**iN-D6 medium**

| **Reagent** | **Final concentration** | **Manufacturer** | **Cat#** |
| --- | --- | --- | --- |
| Neurobasal | 0.5× | Thermo Fisher Scientific | 21103049 |
| B27 with retinoic acid | 1× | Gibco | 17504044 |
| CultureOne supplement | 1× | Gibco | 15674028 |
| L-glutamine | 500 µM | Gibco | 25030-024 |
| Laminin | 1 µg/ml | Sigma | L2020 |
| PenStrep | 100 units/ml | Thermo Fisher Scientific | 15140122 |
| L-ascorbic acid | 200 µM | Sigma | A4403 |
| db-cAMP | 2 mM | Sigma | D0627 |
| NT-3 | 10 ng/ml | Miltenyi Biotec | 130-093-973 |
| BDNF | 10 ng/ml | Miltenyi Biotec | 130-093-811 |
