# Supplementary material for: Alternative splicing coupled to nonsense-mediated decay coordinates downregulation of non-neuronal genes in developing mouse neurons
Source: Genome Biol. 2024 Jun 20;25:162. doi: 10.1186/s13059-024-03305-8 (PMC11188260; doi:10.1186/s13059-024-03305-8)
Supplement: Supplementary file 14 — Additional file 14. Peer review history. [file 13059_2024_3305_MOESM14_ESM.docx]

**Review history**

**First round of review**

**Reviewer 1**

This manuscript provides a detailed characterization of the co-regulation of splicing and transcript abundance during neuron development. In the past, a few alternative splicing (AS) events leading to Nonsense Mediated mRNA Decay (NMD) were described in neurons, but the overall contribution of NMD in these cells was not known because of the difficulty of disentangling changes in transcript levels due to splicing compared to other mechanisms (transcription, degradation etc). The key here is to use Cycloheximide to repress NMD to increase detection of otherwise unstable transcripts. Authors use a new computational tool named factR2 which successfully identified AS-NMD events in their well-validated, inducible neuronal differentiation model. This paper shows that many genes (dozens to low hundred) are regulated by AS-NMD in developing neurons, and that the most common subtype is the poison exons that they named NMD-Stimulating cassette exons (NS-CE). Upon verifying several events, establishing a major role for PTBP1, and deriving the overall expression changes during differentiation, authors show that AS-NMD acts in conjunction with other processes (before, during or after) to dampen the expression of these genes. Last, meta-analysis of other available samples confirms the conserved role of AS-NMD and the prevalence of NS-CE events. I only found one major correction together with minor comments, that need to be satisfactorily addressed before recommending this manuscript for publication.

Major comment:

1. The description of the NMD rules is incomplete and as such, inaccurate (pages 3 and 7): NMD is triggered by premature termination codons (PTC) more than 50 nucleotides upstream of the LAST exon-exon junction, while the definitions on both pages lack the key word "last". This is absolutely crucial, because a PTC in the last five nucleotides of exon 4 of a 10-exon gene (with the translation start in exon 1 and the natural stop in exon 10) should trigger NMD according to the correct rule (because several EJCs are still located downstream of the PTC after the pioneer round of translation). However this case would be incorrectly missed if the pipeline does not follow the correct definition. I seek an honest clarification whether: (1) the definition was wrong but the pipeline for NMD-triggering events was correct (no further action then than changing the two definitions); or (2) the pipeline was wrong. In the second case, the pipeline needs to be corrected and the data needs reanalysis where necessary. Despite that I do not expect the major findings to change at all, this would be essential to prevent releasing a potentially flawed dataset.

Minor comments:

2. In Results, some of the sections need a better explanation of the nature of the data. For instance, the passages on PTBP1 on page 12, what are the datasets for PTBP1 and 1/2 data? I presume these are RNA-seq datasets, but needs explaining and not only providing a reference. Same for the dentate gyrus granule cells on page 17, what is the data?? What I understand is that authors are applying factR2 on previous datasets, so this needs to be made clear.
3. The minigene analysis in Figures 3F-J is good, except that there are several PTBP1 iCLIP tags that are ignored. Authors mutate the two iCLIP tags in the NS exon and one downstream, while there are others actually closer to the NS exon. Please explain why these were not analysed.
4. The expression profile trajectories in Figure S5 are interesting. It would be best to indicate the number of cases (N) following each profile on top of each panel, so we know which ones are more common.
5. Were the CRISPR clones in Figure S13 tested for splicing by RT-PCR? If not, this is necessary. Some exonic fragments are still there after gene editing so it would be possible that they activated a cryptic splice site leading to inclusion of a different cassette exon, hence the interpretation of results might be different. I hope that this was not the case, but we need to know for sure.
6. While the language is good overall, some unclear and vague statements should be fixed. For instance, page 8 "Cassette exons stimulating NMD upon their inclusion (NS-CEs) fared particularly well in this analysis", in which the underlined would be best stated as "were more abundant than the others". Another unclear statement on page 9 needs fixing: "Since NMD occurs in the cytoplasm [12-14], transcripts including NMD-stimulating and excluding NMD-repressing events should be enriched in the nucleus, providing a CHX independent validation approach".

**Reviewer 2**

In this study, Zhuravskaya, Makeyev and colleagues present a transcriptomic analysis of differentiating neurons to estimate the extent to which AS-coupled NMD gene regulation in neurogenesis. That NMD has key roles in this process is well-known, however this study brings to the table a systematic, genome-wide assessment of which genes are regulated by this process. There is potential to discover novel genes and mechanisms from this interesting longitudinal analysis of gene expression.
While my overall impression is favorable, there are some concerns which may require repeated and/or additional analyses, and better discussion. Also the presentation could be improved in some parts.

1. Regarding the association test beween AS-NMD and gene expression levels (page 10, Fig 2), if indeed NMD that they detect regulates gene expression as predicted by their model, we'd expect a strong overlap between genes detected to undergo AS-NMD and genes identified as differentially expressed. Yet the overlap is somewhat modest. They present it as an enrichment in Fig 2B, however the 2-fold to 3-fold enrichment over random overlap is not too impressive: if I read it right, many (a third or a quarter) of the AS-NMD genes are not differentially expressed.

Thus the stringency settings for calling AS-NMD may need to be adjusted and/or pipeline components optimized such as to obtain a better agreement here. Could the results of downstream analysis (functional enrichment etc) be affected by this? They should also keep in mind this dataset will be used as a resource by others thus there would need to be good QC performed.

2. There seems to be some focus in the manuscript on their tool „factR2", which seems to be presented as a novel contribution of their study (at least the version 2 of the tool). If so, there should be a comparison to existing tools which can peform related functions, at the very minimum in a table overview of the functions of factR2 and other tool(s). It would be helpful to have this assessed in terms of analysis outputs on their longitudinal dataset; since they describe in the Discussion that a contribution of their paper is the workflow for identifying AS-NMD (helpful, important) there should be a more detailed comparative analysis of this part of the study.

3. The clustering of trajectories into 28 (!) types. Is the high number of clusters statistically supported (given the number of time points is not that large, and given uncertainty in the estimates)? Are most of them at all biologically meaningful? Most of their analyses seem to use only the 2 extreme types of trajectories if I understand correctly.

4. I do not understand how in Fig 1C and 1D both the NR-CE and the NS-CE cause increased NMD (as evident in increased % of CHX-regulated plasmids). Do they „flip" the sign between NR-CE and NS-CE before the display, so that this shows preferential inclusion of NS-CE and preferetial exclusion of NR-CE (this is not clear from the figure legend?) If this is not the case, then the result would be worrying since it would suggest that predicted NMD-repressing events (NR-CE) in fact promote NMD (and thus that either the prediction is often wrong, or the CHX based measurement).

5. A good part of the study is about the roles of PTBP1 in the AS-NMD, and their dataset offers nice possibilities to identify genes under PTBP1 control e.g. via coexpression analysis or via genetic perturbation. What was less clear from the text which part of these identified genes are actually novel, compared to either „Previous studies by our group and others identified several AS-NMD events controlled by PTBP1, an RNA-binding protein expressed at high levels in ESCs and NPCs and downregulated in neurons [28-31, 34]", or to other knowledge about PTBP1 targets. If they claim „These data suggest that PTBP1 controls a wider range of NS-CEs than previously thought" let us have quantification for that claim. For the newly-identified genes here (including the Fmnl3 and Iqgap1 and Ripk1 that were followed up), how are the confidence scores from their analysis compared to previously well-known target genes of PTBP1 AS-NMD?

Minor:

6. It is curious that „as well as 5881 additional transcripts that had not been previously flagged as possible NMD targets" - can they comment which features their tool can discover that previous tools cannot?

7. The heading „NS-CEs work in coordination with NMD-independent gene downregulation mechanisms" suggests a general claim however that part of the manuscript seems to focus only on the 3 selected genes. Their existing data might be useful to test if this claim holds generally.

8. Overall the manuscript is dense, and loaded with jargon which might be simplified, and with numeric estimates/statistics which might be better simply shown on figures; there is also a bit of an acronym overload. Consider simplifying to make text more accessible to broad audience.

**Reviewer 3**

AS NMD in a developmental paradigm focusing on a forward programming of ESCs into neurons

Overall I like this paper. It is well conceived and address an important issue. The experiments are thoughtfully designed and would add value to the field. I feel it is a strong candidate to be published in GB after some consideration is given to the following points:

Model
1) please can the authors define the neuronal subtype?
2) What is their axial level / regional identity?
3) How enriched are the cultures for neuron subtype specific markers at the protein level? (I appreciate the MuSiC analysis, but it is only transcript level and we know that the relationship between transcript and protein is non linear)
4) Are the neurons functional?
5) How close are these to the regional identity / maturity of then primary cortical cultures used later in this study?

Bioinformatics -
6) tools to predict NMD are not reliable (i.e. poor overlap between those predicted and those identified by experiments). The superiority of the approach employed here could be articulated more definitively.
7) Can the authors comment on their choice of splicing tool in terms of advantages over others (rMATs, MAGIQ etc)

Tools
8) CHX is a non specific tool to inhibit NMD - orthogonality is required e.g. UPF1 KD and/or SMG7 KD, overlapping identified targets.

Discussion
9) can the authors expand their discussion on specific non NMD forms of RNA decay and their relevance in this context
10) Can the authors speculate on how uniform this model would be across different neuronal subtypes, glia and other tissues (I appreciated very much the comparison to granulocytes and agree this argues for broad relevance)
11) how is the mechanism fine tuned in a context specific manner?

Figures:
12) What is the structure shown in S1 D? This 'ball and chain' apprearance suggests the cell bodies have formed 3D clusters with 'fascicles' protruding. This makes analysis esp. omit analysis challenging as it adds the confounding variable of a change in cell-cell interactions - please can the authors comment on this. There are solutions to maintain the cultures as a monolayer e.g. using a cationic substrate like PEI
13) Some poor resolution like S1 E Tubb3
14. Cortical neurons in S4 C look very immature (morphologically).

**Authors’ response to reviewers**

# Reviewer #1

This manuscript provides a detailed characterization of the co-regulation of splicing and transcript abundance during neuron development. In the past, a few alternative splicing (AS) events leading to Nonsense Mediated mRNA Decay (NMD) were described in neurons, but the overall contribution of NMD in these cells was not known because of the difficulty of disentangling changes in transcript levels due to splicing compared to other mechanisms (transcription, degradation etc). The key here is to use Cycloheximide to repress NMD to increase detection of otherwise unstable transcripts. Authors use a new computational tool named factR2 which successfully identified AS-NMD events in their well-validated, inducible neuronal differentiation model. This paper shows that many genes (dozens to low hundred) are regulated by AS-NMD in developing neurons, and that the most common subtype is the poison exons that they named NMD-Stimulating cassette exons (NS-CE). Upon verifying several events, establishing a major role for PTBP1, and deriving the overall expression changes during differentiation, authors show that AS-NMD acts in conjunction with other processes (before, during or after) to dampen the expression of these genes. Last, meta-analysis of other available samples confirms the conserved role of AS-NMD and the prevalence of NS-CE events. I only found one major correction together with minor comments, that need to be satisfactorily addressed before recommending this manuscript for publication.

We thank the reviewer for the positive assessment of our work. Major comment:

1. The description of the NMD rules is incomplete and as such, inaccurate (pages 3 and 7): NMD is triggered by premature termination codons (PTC) more than 50 nucleotides upstream of the LAST exon-exon junction, while the definitions on both pages lack the key word "last". This is absolutely crucial, because a PTC in the last five nucleotides of exon 4 of a 10-exon gene (with the translation start in exon 1 and the natural stop in exon 10) should trigger NMD according to the correct rule (because several EJCs are still located downstream of the PTC after the pioneer round of translation). However this case would be incorrectly missed if the pipeline does not follow the correct definition. I seek an honest clarification whether: (1) the definition was wrong but the pipeline for NMD-triggering events was correct (no further action then than changing the two definitions); or (2) the pipeline was wrong. In the second case, the pipeline needs to be corrected and the data needs reanalysis where necessary. Despite that I do not expect the major findings to change at all, this would be essential to prevent releasing a potentially flawed dataset.

Our pipeline identifies NMD-sensitive candidates as those containing a PTC >50 nt upstream of **the last** exon-exon junction. We have corrected the description on **pages 3 and 7** accordingly.

Minor comments:

2. In Results, some of the sections need a better explanation of the nature of the data. For instance, the passages on PTBP1 on page 12, what are the datasets for PTBP1 and 1/2 data? I presume these are RNA-seq datasets, but needs explaining and not only providing a reference. Same for the dentate gyrus granule cells on page 17, what is the data?? What I understand is that authors are applying factR2 on previous datasets, so this needs to be made clear.

We have clarified these points in the revised text as follows:

**Page 12**. "To identify PTBP1-dependent AS-NMD targets systematically, we examined factR2 events correlating with Ptbp1 expression in DMSO-treated TRE- Ngn2 cells and changing their splicing in ESCs or NPCs in response to PTBP1 knockdown alone or together with its functionally similar paralog, PTBP2 (Fig. 3A; RNA-seq data from [56]; Whippet |ΔPSI|>0.1 and probability>0.9 cutoffs; see Materials and Methods for further details)"

**Page 17**. "To examine the incidence of NS-CEs in a different system, we performed factR2 analysis for developing dentate gyrus granule cells (RNA-seq data from [60])..."

3. The minigene analysis in Figures 3F-J is good, except that there are several PTBP1 iCLIP tags that are ignored. Authors mutate the two iCLIP tags in the NS exon and one downstream, while there are others actually closer to the NS exon. Please explain why these were not analysed.

Putative PTBP1 sites shown in **Fig. 3F** are pyrimidine-rich sequence motifs, not iCLIP clusters. As shown in **Fig. S11A**, there are only 2 intronic positions where such pyrimidine-rich sequences correspond to a single PTBP1 iCLIP cluster. We therefore designed mut3 to inactivate these iCLIP-overlapping motifs. Mut1 and mut2 were designed to inactivate sequence motifs within the regulated Fmnl3 exon. We have revised **Fig. 3F** and **Fig. S11** for clarity.

4. The expression profile trajectories in Figure S5 are interesting. It would be best to indicate the number of cases (N) following each profile on top of each panel, so we know which ones are more common.

Done (the Figure is now called **S6**).

5. Were the CRISPR clones in Figure S13 tested for splicing by RT-PCR? If not, this is necessary. Some exonic fragments are still there after gene editing so it would be possible that they activated a cryptic splice site leading to inclusion of a different cassette exon, hence the interpretation of results might be different. I hope that this was not the case, but we need to know for sure.

This experiment is presented in the revised **Fig. S15**. Of the 6 mutant clones tested, only A15 and E2 show detectable inclusion of cryptic exons. However, even in these cases, the cryptic exons are included with markedly lower efficiencies compared to the wild-type NS-CEs. Together with the data in **Fig. 5G-I**, this suggests that the mutations disrupted the AS-NMD regulation.

6. While the language is good overall, some unclear and vague statements should be fixed. For instance, page 8 "Cassette exons stimulating NMD upon their inclusion (NS-CEs) fared particularly well in this analysis", in which the underlined would be best stated as "were more abundant than the others". Another unclear statement on page 9 needs fixing: "Since NMD occurs in the cytoplasm [12-14], transcripts including NMD-stimulating and excluding NMD-repressing events should be enriched in the nucleus, providing a CHX independent validation approach".

We have modified the unclear sentences as follows:

**Page 8**. "Cassette exons stimulating NMD upon their inclusion (NS-CEs) were associated with particularly robust regulation in neural cells (days 3-24; Fig. 1C)."

**Page 9**. "Since NMD occurs in the cytoplasm [12-14], transcripts including NMD- stimulating and excluding NMD-repressing events are expected to be enriched in the nucleus. We reasoned that such nuclear enrichment could provide a CHX-independent validation method for AS-NMD targets."

# Reviewer #2

In this study, Zhuravskaya, Makeyev and colleagues present a transcriptomic analysis of differentiating neurons to estimate the extent to which AS-coupled NMD gene regulation in neurogenesis. That NMD has key roles in this process is well-known, however this study brings to the table a systematic, genome-wide assessment of which genes are regulated by this process. There is potential to discover novel genes and mechanisms from this interesting longitudinal analysis of gene expression.

While my overall impression is favorable, there are some concerns which may require repeated and/or additional analyses, and better discussion. Also the presentation could be improved in some parts.

1. Regarding the association test beween AS-NMD and gene expression levels (page 10, Fig 2), if indeed NMD that they detect regulates gene expression as predicted by their model, we'd expect a strong overlap between genes detected to undergo AS- NMD and genes identified as differentially expressed. Yet the overlap is somewhat modest. They present it as an enrichment in Fig 2B, however the 2-fold to 3-fold enrichment over random overlap is not too impressive: if I read it right, many (a third or a quarter) of the AS-NMD genes are not differentially expressed.

Thus the stringency settings for calling AS-NMD may need to be adjusted and/or pipeline components optimized such as to obtain a better agreement here. Could the results of downstream analysis (functional enrichment etc) be affected by this? They should also keep in mind this dataset will be used as a resource by others thus there would need to be good QC performed.

We thank the reviewer for raising this point. The analysis in **Fig. 2** was carried out to shortlist AS-NMD events that might **drive gene expression changes or facilitate such changes initiated by other mechanisms** (i.e. transcription). However, not all events are expected to work in this manner. For instance, AS-NMD often **stabilizes expression** of genes encoding RNA-binding proteins (RBPs) via negative feedback. This may be the reason why we detect previously described AS-NMD events in RBP genes such as *Srsf11/p54*, *Tra2a* or *Tra2b* [PMIDs 14709600, 17361132, and 17369403] as CHX-responsive (revised **Table S2**) but not "facilitating" (revised Table S4).

We used reasonably stringent |ΔPSI|>0.1 and probability>0.9 Whippet cutoffs throughout our study. In our experience, this allows sufficiently sensitive detection of true positive AS events. Increasing the cutoff stringency to |ΔPSI|>0.25 and probability>0.95 improved the overlap between AS and gene regulation (new **Fig. S5**). However, this also resulted in the loss of some true-positive events, including

e.g. the NMD-stimulating cassette exon in the *Srsf9* gene validated in **Fig. S9** or the NMD-stimulating alternative donor in *Hps1*, which we characterized previously [PMID: 25375251].

Thus, our current settings appear to strike a reasonable balance between stringency and sensitivity. Nonetheless, we added the more stringently defined AS-NMD targets as the second tab in the revised **Table S4**, keeping in mind that our data might be used as a resource for others.

2. There seems to be some focus in the manuscript on their tool „factR2", which seems to be presented as a novel contribution of their study (at least the version 2 of the tool). If so, there should be a comparison to existing tools which can peform related functions, at the very minimum in a table overview of the functions of factR2 and other tool(s). It would be helpful to have this assessed in terms of analysis outputs on their longitudinal dataset; since they describe in the Discussion that a contribution of their paper is the workflow for identifying AS-NMD (helpful, important) there should be a more detailed comparative analysis of this part of the study.

As suggested, we added a table (new **Table S1**) showing that factR2 provides a wider range of relevant functions compared to other tools for AS-NMD analyses.

3. The clustering of trajectories into 28 (!) types. Is the high number of clusters statistically supported (given the number of time points is not that large, and given uncertainty in the estimates)? Are most of them at all biologically meaningful? Most of their analyses seem to use only the 2 extreme types of trajectories if I understand correctly.

We consistently obtain 28 clusters using the DP_GP_cluster program, which is designed	for	longitudinal	gene	expression	trajectories (https://github.com/PrincetonUniversity/DP_GP_cluster). Conceptually, each transition from a given differentiation stage to the next one may involve 3 possible gene regulation scenarios: no change, upregulation, or downregulation. Considering a time course with 5 time points connected by 4 transitions, the maximum number of possibilities is 3^4=81. Thus, we have no reason to believe that 28 clusters overfit the data.

To explore the effect of cluster number systematically, we grouped expression trajectories using the k-means method. This argued that NS-CE events are significantly enriched in neurodevelopmentally downregulated genes **regardless of the k value** (new **Fig. S7**). Therefore, the number of clusters does not affect our original conclusion.

4. I do not understand how in Fig 1C and 1D both the NR-CE and the NS-CE cause increased NMD (as evident in increased % of CHX-regulated plasmids). Do they

„flip" the sign between NR-CE and NS-CE before the display, so that this shows preferential inclusion of NS-CE and preferetial exclusion of NR-CE (**this is not clear from the figure legend?**) If this is not the case, then the result would be worrying since it would suggest that predicted NMD-repressing events (NR-CE) in fact promote NMD (and thus that either the prediction is often wrong, or the CHX based measurement).

**Fig. 1C-D** illustrate the magnitude of CHX-induced gene upregulation for different groups of AS-NMD targets responding to CHX at the splicing level. As described on **page 8** of the revised manuscript, we indeed "flipped the sign" for the splicing regulation in the following way:

"We used Whippet [51] for the splicing-level analysis and selected CEs, ADs, AAs and RIs consistently changing their percent spliced in (PSI) values in response to CHX at least at one differentiation stage (NS events, ΔPSI>0.1; NR events, ΔPSI<- 0.1; probability>0.9). We additionally selected NS events with a PSI average of >0.9 for DMSO- and CHX-treated samples, and NR events with a PSI average of <0.1. This was done because these groups have a limited capacity to change splicing in response to CHX but may still be regulated at the gene expression level."

We now refer to this in-text description in the **Fig. 1** legend.

5. A good part of the study is about the roles of PTBP1 in the AS-NMD, and their dataset offers nice possibilities to identify genes under PTBP1 control e.g. via coexpression analysis or via genetic perturbation. What was less clear from the text which part of these identified genes are actually novel, compared to either „Previous studies by our group and others identified several AS-NMD events controlled by PTBP1, an RNA-binding protein expressed at high levels in ESCs and NPCs and downregulated in neurons [28-31, 34]", or to other knowledge about PTBP1 targets. If they claim „These data suggest that PTBP1 controls a wider range of NS-CEs than previously thought" let us have quantification for that claim. For the newly-identified genes here (including the Fmnl3 and Iqgap1 and Ripk1 that were followed up), how are the confidence scores from their analysis compared to previously well-known target genes of PTBP1 AS-NMD?

A PubMed search for the **46** PTBP1-regulated events in revised **Table S4** (non-n/a values in the "Predicted effect of PTBP1 on AS-NMD event" column) showed that only **8** of them had been previously characterized as PTBP1 targets. We added the corresponding PMID or "None" values to the "PubMed results for PTBP1 regulation" column of **Table S4**. Incidentally, this analysis confirmed our original description, which we left unchanged in the revised manuscript:

**Page 13**. "PTBP1 was proposed to control Ripk1 and Iqgap1 NS-CE splicing [27, 57- 59], but the role of these exons in the regulation of their host genes during neuronal differentiation was not addressed experimentally. To the best of our knowledge, the Fmnl3 NS-CEs has not been previously identified."

We hope the new column in **Table S4** answers Reviewer's question. However, we are reluctant (and probably not allowed by the journal) to claim that the **38** PTBP1 targets not found on PubMed are "novel". It is possible that some of them are mentioned in published supplemental data, which are not searched by PubMed, etc. With this in mind, we do not think it would be informative to compare confidence scores between the two groups of targets.

Minor:

6. It is curious that „as well as 5881 additional transcripts that had not been previously flagged as possible NMD targets" - can they comment which features their tool can discover that previous tools cannot?

factR2 categorizes transcripts based on their gene of origin and supplements unannotated transcripts with open reading frame (ORF) information derived from known protein-coding isoforms. The program then consistently applies the >50 nt from a stop codon to the last exon junction rule to shortlist NMD candidates.

We introduced a new panel (**Fig. S3A**) displaying the GENCODE biotypes for factR2-predicted NMD targets. The new NMD candidates come from four biotypes: protein_coding, retained_intron, processed_transcript, and lncRNA. In the protein_coding group, factR2 tends to identify a premature termination codon (PTC) overlooked by GENCODE. For the remaining three groups, NMD sensitivity is typically predicted by detecting a PTC in the newly annotated ORF.

7. The heading „NS-CEs work in coordination with NMD-independent gene downregulation mechanisms" suggests a general claim however that part of the manuscript seems to focus only on the 3 selected genes. Their existing data might be useful to test if this claim holds generally.

To find out if NS-CEs may cooperate with NMD-independent mechanisms globally, we examined developmental trajectories of genes containing CHX-responsive facilitating NS-CEs with and without CHX (new **Fig. S16**). Here is the description of this analysis in the revised manuscript:

**Page 17**. "Our additional bioinformatics analysis showed that genes containing functional NMD-stimulated cassette exons generally maintained declining neurodevelopmental trends in the presence of CHX, even though their downregulation was more robust in DMSO- compared to CHX-treated samples (Fig. S16). This points to a wider crosstalk between AS-NMD and other gene regulation mechanisms."

8. Overall the manuscript is dense, and loaded with jargon which might be simplified, and with numeric estimates/statistics which might be better simply shown on figures; there is also a bit of an acronym overload. Consider simplifying to make text more accessible to broad audience.

We have edited the text for clarity and removed numeric estimates/statistics and acronyms, where possible.

# Reviewer #3

AS NMD in a developmental paradigm focusing on a forward programming of ESCs into neurons

Overall I like this paper. It is well conceived and address an important issue. The experiments are thoughtfully designed and would add value to the field.

Thank you very much for pointing this out.

I feel it is a strong candidate to be published in GB after some consideration is given to the following points:

Model

1) please can the authors define the neuronal subtype?
2) What is their axial level / regional identity?

To address these questions, we conducted subtype/region-specific MuSiC analysis. Alongside the cell type deconvolution in the **Fig. S2C**, this new data suggest that induced TRE-Ngn2 neurons tend to share gene expression patterns with several groups of glutamatergic cortical/hippocampal neurons (new **Fig. S2D**). The prominence of three distinct axial/regional signatures at the final differentiation time point (Cajal-Retzius, L5 pyramidal-tract cortical, and CA3 hippocampal neurons) suggests that the TRE-Ngn2 glutamatergic neurons have heterogeneous or mixed identities (**Fig. S2D**). This is not unusual for in vitro differentiation systems – for example, NGN2-induced human neurons are likely even more heterogeneous [e.g., PMID: 34358451].

3) How enriched are the cultures for neuron subtype specific markers at the protein level? (I appreciate the MuSiC analysis, but it is only transcript level and we know that the relationship between transcript and protein is non linear).

We have extended our immunofluorescence analyses by co-staining mature induced neurons for pre- and post-synaptic markers (synaptophysin/SYP and DLG4/PSD-95). This produced characteristic punctate staining patterns (new **Fig. S1F**). Combined with the TUBB3/TUJ1, MAP2, and ANK3/ANKG immunofluorescence data in **Fig. S1C-E**, this argues that the upregulation of the corresponding mRNAs in differentiating TRE-Ngn2 cells (**Fig. S2B** and **Fig. S10A**) is mirrored at the protein level.

4) Are the neurons functional?

The current set of our immunostaining data shows that the induced neurons express both pre- and post-synaptic markers (SYP and DLG4), as well as the axon initial segment marker ANK3 (**Fig. S1D-F**). Interestingly, we often detect SYP- and DLG4- positive puncta near each other, pointing to possible synapse formation (see close-ups in the newly added panel **Fig. S1F**). We believe that more involved tests for neuronal functionality would go outside of the scope of our study, which is focused on a gene regulation mechanism.

How close are these to the regional identity / maturity of then primary cortical cultures used later in this study?

Correlating gene expression between different samples (new **Fig. S4D**) suggests that the day-in-vitro 5 (DIV5) and day-in-vitro 21 (DIV21) cortical neurons used in our work correspond to the differentiation day 12 and 24 TRE-Ngn2 neurons, respectively.

Bioinformatics -

5) tools to predict NMD are not reliable (i.e. poor overlap between those predicted and those identified by experiments). The superiority of the approach employed here could be articulated more definitively.

We have added a new table (**Table S1**) highlighting the improvements offered by factR compared to other currently available packages.

6) Can the authors comment on their choice of splicing tool in terms of advantages over others (rMATs, MAGIQ etc).

We selected Whippet because it is fast and has very modest memory requirements. We could not use MAJIQ because it focuses on **splice junctions**, while we wanted to identify NMD-associated **events**. To check if the choice of splicing tools matters, we reanalyzed our data by the event-centric program rMATS. The new result (**Fig. S3D**) closely resembles the original Whippet-produced panel (**Fig. 1E**), confirming the robustness of our AS-NMD event shortlisting procedure.

Tools

7) CHX is a non specific tool to inhibit NMD - orthogonality is required e.g. UPF1 KD and/or SMG7 KD, overlapping identified targets.

We analyzed the effect of UPF1 knockdown for the three shortlisted targets (Fmnl3, Iqgap1 and Ripk1; revised **Fig. S12**). Although the knockdown of UPF1 was only partial (**Fig. S12A**), we detected statistically significant rescue of all three targets in siPtbp1-treated samples (**Fig. S12B-D**).

Discussion

8) can the authors expand their discussion on specific non NMD forms of RNA decay and their relevance in this context.

To address this comment, we have added the following sentence with some relevant references on **page 22**: "Exploring potential crosstalk between AS-NMD and these, as well as other processes regulating mRNA stability, nucleocytoplasmic distribution, and translational activity [77-79] in different developmental and physiological contexts may unveil novel mechanisms regulating eukaryotic gene expression."

9) Can the authors speculate on how uniform this model would be across different neuronal subtypes, glia and other tissues (I appreciated very much the comparison to granulocytes and agree this argues for broad relevance).

We have characterized dentate gyrus granule cells, as an example of a distinct group of neurons (**pages 17-18**; **Fig. 6A-D**). This analysis identified numerous AS-NMD targets that partially overlapped their TRE-Ngn2 counterparts (revised **Table S6**). A separate study will be required for systematic comparisons of AS-NMD targets across different tissues and cell types.

10) how	is	the	mechanism	fine	tuned	in	a	context	specific	manner?

The revised Discussion alludes to potential RPB-based mechanisms, as well as the crosstalk between AS-NMD and other post-transcriptional processes (see **pages 21- 22**). We feel that a more elaborate discussion would be too speculative at this stage.

Figures:

11) What is the structure shown in S1 D? This 'ball and chain' apprearance suggests the cell bodies have formed 3D clusters with 'fascicles' protruding. This makes analysis esp. omit analysis challenging as it adds the confounding variable of a change in cell-cell interactions - please can the authors comment on this. There are solutions to maintain the cultures as a monolayer e.g. using a cationic substrate like PEI

**Fig. S1D** shows an individual neuron, and we have now added DAPI staining for clarity. In our procedure, surfaces are coated with diluted Geltrex to minimize clustering of neuronal somas and fasciculation of neurites (see Materials and Methods).

12) Some poor resolution like S1 E Tubb3

We now show a better-resolved image in **Fig. S1E**.

14. Cortical neurons in S4 C look very immature (morphologically).

The original version of **Fig. S4C** displayed DIV5 neurons stained for the neuronal marker MAP2. We have added MAP2-staining data for DIV21 to the revised **Fig. S4C**, where the neurons have more mature appearance.

**Second round of review**

**Reviewer 1**

All ok now.

**Reviewer 2**

In the revised manuscript, the authors have adressed issues raised by myself and other reviewers.

I have outstanding comments that need to be resolved, regarding author responses to my queries.

I think their answer to point 1 is lacking. A lack of correlation bewteen the estimated AS-NMD and the changes in gene expression levels might indeed be explained by feedback mechanisms as they write. However this is besides the point. The burden of proof is on them to show these regulatory changes driven by AS-NMD do occur and can be measured robustly, instead of finding complicated explanation why they are difficult to measure and claiming that this is evidence they must nonetheless exist (it is not evidence thereof). The lack of agreement/low enrichments are more likely explained by measurement error, which could be substantial.

If their measurements of AS-NMD have certain accuracy and make some non-negligible amount of error (which seems to be the case judging from above), they should state this the text with more transparency and caution to the reader: please state clearly in the text what are the enrichments regarding "CHX-responsive AS-NMD events were enriched for positively correlated entries (Fig. 2A). The enrichment was predominantly due to CEs rather than other types of AS-NMD events (Fig. 2A-B)." Moreover please in addition to the p-value state also the FDR for the "Hereafter, we will use the term "facilitating" to refer to CHX-responsive events showing significant positive correlation (P<0.05) between NMD-protective splicing patterns and gene expression." and related in Table S4 the column "AS-NMD event-gene correlation, P-value" should also have a FDR corrected corresponding column in the same table, in both sheets. (probable typo: confusingly one sheet states Table S4 and other Table S3).

Their answer to the trajectory clustering concern "the maximum number of possibilities is 3^4=81. Thus, we have no reason to believe that 28 clusters overfit the data." actually argues for the opposite of what they think it argues. 28 clusters out of 81 possible single-instance clusters is a very high number of clusters. How well this is supported in the data or how it helps interpretation is a little unclear. I do understand and agree that this clustering is not the most critical point for the conclusion, but it sticks out not in a good way. It would improve the manuscript to add a caveat.

Regarding my last comment "Overall the manuscript is dense, and loaded with jargon which might be simplified..." it could have been addressed much better than it was in this revision. This is obviously just a presentation issue and does not change the quality/interest of the results, but I do think it is worth to invest much more effort to make the manuscript accessible to a broad audience. Also, consider making it shorter by relegating parts to a supplementary text.

**Authors’ response to reviewers**

Reviewer #2:

In the revised manuscript, the authors have adressed issues raised by myself and other

reviewers.

I have outstanding comments that need to be resolved, regarding author responses to my

queries.

I think their answer to point 1 is lacking. A lack of correlation bewteen the estimated AS-NMD and the changes in gene expression levels might indeed be explained by feedback mechanisms as they write. However this is besides the point. The burden of proof is on them to show these regulatory changes driven by AS-NMD do occur and can be measured robustly, instead of finding complicated explanation why they are difficult to measure and claiming that this is evidence they must nonetheless exist (it is not evidence thereof). The lack of agreement/low enrichments are more likely explained by measurement error, which could be substantial.

If their measurements of AS-NMD have certain accuracy and make some non-negligible amount of error (which seems to be the case judging from above), they should state this the text with more transparency and caution to the reader: please state clearly in the text what are the enrichments regarding "CHX-responsive AS-NMD events were enriched for positively correlated entries (Fig. 2A). The enrichment was predominantly due to CEs rather than other types of AS-NMD events (Fig. 2A-B)."

We agree that measurement errors might underlie the lack of detectable correlation of some AS-NMD events with gene expression dynamics. Indeed, we see an increase in correlation when we consider more stringently defined, i.e. less "noisy" groups of NS-CE events. Compared to control events, NS-CEs shortlisted with ΔPSI>0.1 and probability>0.9 cutoffs were enriched for significantly correlated entries **~2.4-fold** (Fig. 2A-B; correlation P<0.05), whereas NS-CEs shortlisted with ΔPSI>0.25 and probability>0.95 cutoffs were enriched for significantly correlated entries **~2.7-fold** (Fig. S5). An even stronger gain in enrichment was detected for the more stringent correlation *P*-value of 0.001: **~3.8-fold** for ΔPSI>0.1 and probability>0.9 NS-CEs (Fig. 2A-B) and **~5.8-fold** for the ΔPSI>0.25 and probability>0.95 NS-CEs (Fig. S5).

As suggested by the reviewer, we now explicitly mention fold enrichment values in the

Results (page 10): "The enrichment of correlated events amongst NS-CEs ranged from

~2.4-fold (ΔPSI>0.1 and probability>0.9 Whippet cutoffs and P<0.05 correlation cutoff; Fig. 2A-B) to ~5.8-fold (ΔPSI>0.25 and probability>0.95 Whippet cutoffs and P<0.001 correlation cutoff; Fig. S5)."

For further transparency, we have included the following paragraph in the revised Discussion (page 21): "It should be noted that we utilized relatively lenient cutoffs (Whippet ΔPSI>0.1, probability>0.9 and correlation P<0.05) to shortlist AS-NMD events potentially influencing gene expression in neurodevelopment (the first tab in Table S4). factR2 users may consider adjusting these parameters, based on the data in Fig. 2A-B, Fig. S5, and the second tab of Table S4, to strike the optimal balance between capturing all regulatory events and events with the strongest regulation potential."

Moreover please in addition to the p-value state also the FDR for the "Hereafter, we will use the term "facilitating" to refer to CHX-responsive events showing significant positive correlation (P<0.05) between NMD-protective splicing patterns and gene expression." And related in Table S4 the column "AS-NMD event-gene correlation, P-value" should also have a FDR corrected corresponding column in the same table, in both sheets. (probable typo: confusingly one sheet states Table S4 and other Table S3).

We have added the FDR column in both tabs of Table S4 and now mention the FDR cutoff in the revised text: "Hereafter, we will use the term "facilitating" to refer to CHX-responsive events showing significant positive correlation (P<0.05; FDR<0.1) between NMD-protective splicing patterns and gene expression". We have also corrected the typo in the title of the second tab of Table S4.

Their answer to the trajectory clustering concern "the maximum number of possibilities is 3^4=81. Thus, we have no reason to believe that 28 clusters overfit the data." actually argues for the opposite of what they think it argues. 28 clusters out of 81 possible single- instance clusters is a very high number of clusters. How well this is supported in the data or how it helps interpretation is a little unclear. I do understand and agree that this clustering is not the most critical point for the conclusion, but it sticks out not in a good way. It would improve the manuscript to add a caveat.

To address this concern and the suggestion on shortening the manuscript (see below), we moved all the clustering data using both DP_GP_cluster and k-means methods to the supplement (see new Fig. S7 and its legend). Fig. 2 now shows enrichment data for individual genes (panels C-F). Our original conclusion remains unchanged: genes with CHX- responsive facilitating CEs, particularly NS-CEs are enriched for monotonic downregulation in neurodevelopment.

Regarding my last comment "Overall the manuscript is dense, and loaded with jargon which might be simplified..." it could have been addressed much better than it was in this revision. This is obviously just a presentation issue and does not change the quality/interest of the results, but I do think it is worth to invest much more effort to make the manuscript accessible to a broad audience. Also, consider making it shorter by relegating parts to a supplementary text.

As suggested, we have substantially shortened the part of the Results that deals with gene clustering approaches by relegating the clustering data to Fig. S7. To further streamline the manuscript, we moved the last section of the Results, *NS-CEs may contribute to gene downregulation in other developmental contexts*, to the Supplement and simplified several sentences throughout the text. Although it has not been possible to completely eliminate technical terminology, we hope that these changes enhance readability and make the manuscript more accessible.

**Third round of review**

**Reviewer 2**

The authors have satisfactorily addressed my concerns, improving the reporting of QC steps in the analyses, and the presentation of the study by focussing the text. I can recommend publication of this interesting study of NMD in development of neural cells.
